# p300 suppresses the transition of myelodysplastic syndromes to acute myeloid leukemia

**DOI:** 10.1172/jci.insight.138478

**Published:** 2021-10-08

**Authors:** Na Man, Gloria Mas, Daniel L. Karl, Jun Sun, Fan Liu, Qin Yang, Miguel Torres-Martin, Hidehiro Itonaga, Concepcion Martinez, Shi Chen, Ye Xu, Stephanie Duffort, Pierre-Jacques Hamard, Chuan Chen, Beth E. Zucconi, Luisa Cimmino, Feng-Chun Yang, Mingjiang Xu, Philip A. Cole, Maria E. Figueroa, Stephen D. Nimer

**Affiliations:** 1 Sylvester Comprehensive Cancer Center,; 2 Department of Medicine, and; 3 Department of Biochemistry and Molecular Biology, University of Miami Miller School of Medicine, Miami, Florida, USA.; 4 Division of Genetics, Departments of Medicine and Biological Chemistry and Molecular Pharmacology, Harvard Medical School and Brigham & Women’s Hospital, Boston, Massachusetts, USA.; 5 Department of Human Genetics, University of Miami Miller School of Medicine, Miami, Florida, USA.

**Keywords:** Hematology, Epigenetics, Leukemias

## Abstract

Myelodysplastic syndromes (MDS) are hematopoietic stem and progenitor cell (HSPC) malignancies characterized by ineffective hematopoiesis and an increased risk of leukemia transformation. Epigenetic regulators are recurrently mutated in MDS, directly implicating epigenetic dysregulation in MDS pathogenesis. Here, we identified a tumor suppressor role of the acetyltransferase p300 in clinically relevant MDS models driven by mutations in the epigenetic regulators TET2, ASXL1, and SRSF2. The loss of p300 enhanced the proliferation and self-renewal capacity of *Tet2-*deficient HSPCs, resulting in an increased HSPC pool and leukemogenicity in primary and transplantation mouse models. Mechanistically, the loss of p300 in *Tet2-*deficient HSPCs altered enhancer accessibility and the expression of genes associated with differentiation, proliferation, and leukemia development. Particularly, p300 loss led to an increased expression of *Myb*, and the depletion of *Myb* attenuated the proliferation of HSPCs and improved the survival of leukemia-bearing mice. Additionally, we show that chemical inhibition of p300 acetyltransferase activity phenocopied *Ep300* deletion in *Tet2*-deficient HSPCs, whereas activation of p300 activity with a small molecule impaired the self-renewal and leukemogenicity of *Tet2-*deficient cells. This suggests a potential therapeutic application of p300 activators in the treatment of MDS with TET2 inactivating mutations.

## Introduction

Normal hematopoiesis is a process that involves the coordination of stem and progenitor cell self-renewal and differentiation. Hematopoietic cell fate is determined by the patterns of gene expression, which are directed by the chromatin structure and the presence or absence of transcription factors (TFs) and cofactors. The regulation of hematopoiesis requires specific and highly ordered epigenetic programming, including dynamic changes in DNA methylation, histone modifications, and chromatin remodeling. Recently, systematic genome profiling screens have identified recurring epigenetic dysregulation in hematopoietic malignancies (1).

Myelodysplastic syndromes (MDS) are among the most common hematological malignancies, with an increased incidence during aging. MDS are stem cell diseases featured by ineffective hematopoiesis with peripheral blood cytopenias, dysplastic and aberrant hematopoietic cell differentiation, and increased leukemia transformation potential (2). Somatic and germline mutations in MDS patients affect genes encoding DNA methylation regulators (TET2 and DNMT3A), chromatin modifiers (ASXL1 and EZH2), splicing factors (SRSF2, U2AF1, and SF3B1), TFs (EVI1, RUNX1, and GATA2), and signaling pathway intermediates (3). Among these mutations, *TET2* inactivating mutations are found in approximately 20% of MDS patients but are also seen in patients with acute myeloid leukemia (AML) and myeloproliferative neoplasms (4–7). TET2 converts 5-methylcytosine to 5-hydroxymethylcytosine (5-hmC), a key step in the dynamic regulation of DNA methylation. The loss of *Tet2* in hematopoietic cells leads to a global loss of 5-hmC and hematopoietic defects, including enhanced hematopoietic stem cell (HSC) self-renewal, myeloid cell expansion, and an increased propensity to develop MDS or acute leukemia (8–13).

p300, encoded by the *Ep300* gene, is a lysine acetyltransferase (KAT) that plays pivotal roles in cellular proliferation and signal transduction by acting as a transcriptional cofactor and chromatin modifier (14). In normal hematopoietic cells, p300 regulates HSC self-renewal and differentiation (15) and the loss of p300 impairs B and T cell differentiation (16, 17). In malignant hematopoiesis, it is currently unclear whether p300 plays a suppressive or an oncogenic role. For example, in AML1-ETO–driven leukemia, p300 acetylates AML1-ETO to promote leukemia and inhibition of p300 KAT activity impairs leukemia cell growth (18, 19). However, *Ep300* mutations are found in patients with myeloid neoplasia and the low expression level of p300 is a poor prognostic marker (20). Our previous work also identified a tumor suppressor role of p300 in myeloid transformation based on a *Nup98-Hoxd13*–transgenic mouse model of MDS (21, 22). Comutations of *EP300* and *TET2* are found in patients with myeloid neoplasms (23). Moreover, p300 directly acetylates TET2 and these enzymes cooperate to regulate HSC enhancer function and facilitate transcription (24, 25). Yet, the direct molecular and biological effects of p300 deletion or inhibition on HSC homeostasis in the absence of TET2 are still unknown.

Here, we demonstrate that p300 is a general epigenetic suppressor of MDS progression using 4 clinically relevant, genetically engineered MDS mouse models: *Tet2^+/–^*, *Tet2^–/–^*, *Asxl1^+/–^* and *Srsf2^P59H^*. In all models, *Ep300* deletion shortened the survival of the mice and accelerated the development of MDS/AML. We focused primarily on the influence of p300 loss in *Tet2*-deficient hematopoiesis and found that the loss of p300 promoted the self-renewal and proliferation of *Tet2-*deficient hematopoietic stem and progenitor cells (HSPCs), resulting in an increased HSPC pool in the bone marrow and accelerated leukemia transformation. Mechanistically, the loss of p300 in *Tet2-*deficient HSPCs affected the interplay between enhancers and TF networks altering the expression of genes related to differentiation, signal transduction, immune response, cell proliferation, and oncogenicity. In particular, we found that the loss of p300 increased the expression of the proto-oncogene *Myb* in multiple MDS models, identifying a common mechanism underlying the accelerated disease progression seen after p300 loss. Accordingly, the depletion of *Myb* reversed the hyperproliferative phenotype of HSPCs lacking both p300 and TET2. We also show that manipulating p300 catalytic activity by small molecules exacerbated or mitigated *Tet2*-deficient hematologic malignancies.

## Results

### The loss of p300 in Tet2-deficient mouse models shortened survival and accelerated the development of MDS and progression to AML.

To investigate the role of p300 in MDS progression driven by *Tet2* deficiency, we crossed *Tet2*-deficient (*Tet2^–/–^* and *Tet2^+/–^*) mice with hematopoietic-specific conditional knockout *Ep300* mice and generated primary and transplantation mouse models. The deletion of *Ep300* was verified by quantitative RT-PCR in the bone marrow cells 2 weeks after poly(I:C) administration (Supplemental Figure 1, A and B; supplemental material available online with this article; https://doi.org/10.1172/jci.insight.138478DS1).

**Figure 1:**
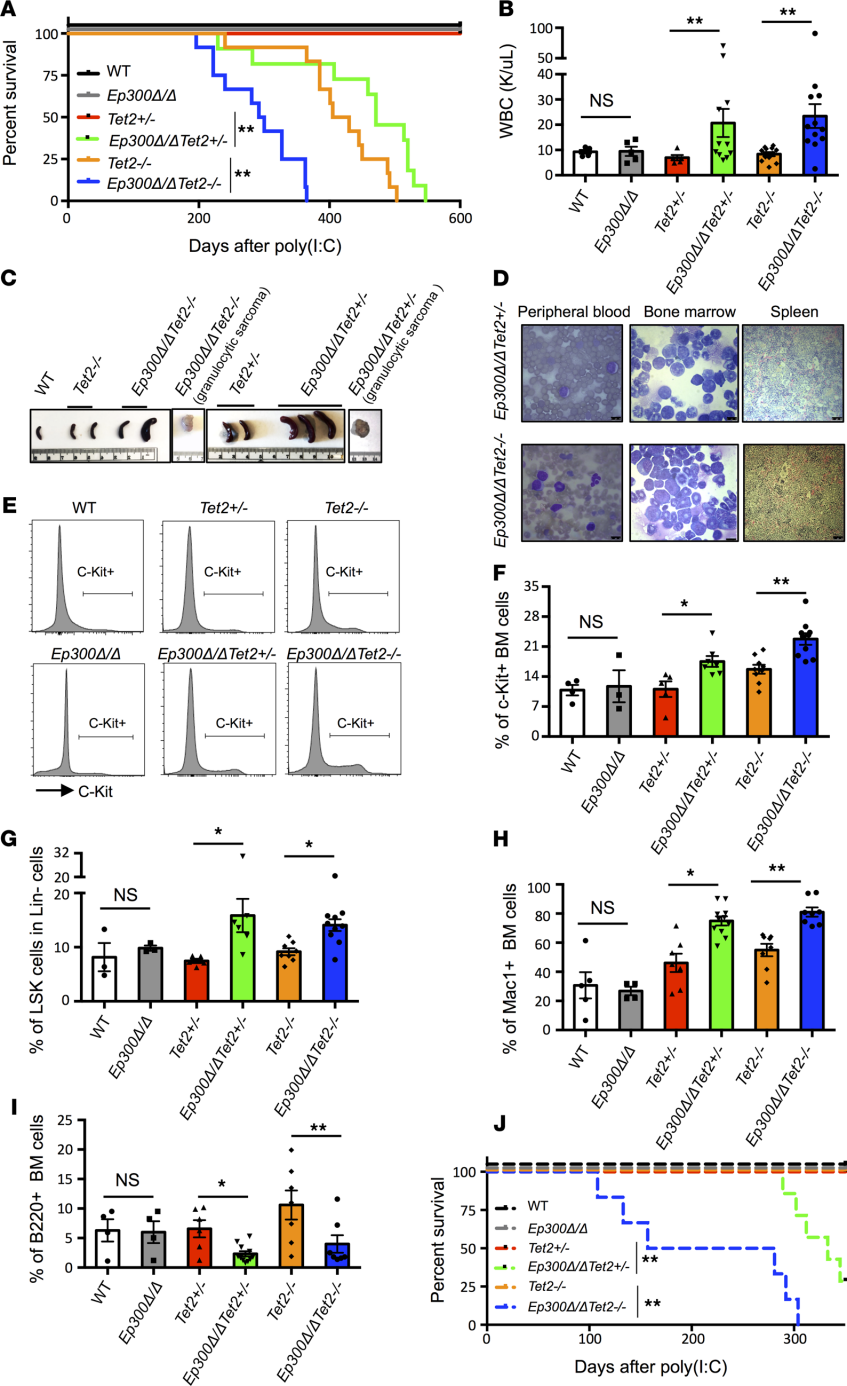
Loss of p300 in Tet2-deficient mice accelerates the onset of leukemia and shortens survival. (**A**) Kaplan-Meier survival curve of mice after poly(I:C) injections for each genotype (WT, *Ep300*Δ*/*Δ, *Tet2^+/–^*, *Ep300*Δ*/*Δ*Tet2^+/–^*, *Tet2^–/–^*, and *Ep300*Δ*/*Δ*Tet2^–/–^*). Loss of p300 in Tet2-deficient mice shortens the survival time. (**B**) WBC counts in peripheral blood of endpoint *Ep300*Δ*/*Δ*Tet2^+/–^* and *Ep300*Δ*/*Δ*Tet2^–/–^* mice and age-matched *Tet2^+/–^* and *Tet2^–/–^* mice. The WT, *Ep300*Δ*/*Δ, and *Tet2^–/–^* mice were age-matched to *Ep300*Δ*/*Δ*Tet2^–/–^* mice and *Tet2^+/–^* mice were age-matched to *Ep300*Δ*/*Δ*Tet2^+/–^* mice. (**C**) Splenomegaly and granulocytic sarcoma found in *Ep300*Δ*/*Δ*Tet2^+/–^* and *Ep300*Δ*/*Δ*Tet2^–/–^* mice at endpoint, compared with age-matched WT, *Tet2^–/–^*, and *Tet2^+/–^* mice. (**D**) Morphology of peripheral blood and bone marrow cells and histology of spleen from moribund *Ep300*Δ*/*Δ*Tet2^+/–^* and *Ep300*Δ*/*Δ*Tet2^–/–^* mice. Scale bar for peripheral blood and bone marrow cells is 10 μm and for spleen is 50 μm. (**E** and **F**) Representative flow cytometry profiles (**E**) and percentage of c-Kit^+^ cells in the bone marrow (**F**) of moribund *Ep300*Δ*/*Δ*Tet2^+/–^* and *Ep300*Δ*/*Δ*Tet2^–/–^* mice and age-matched *Tet2^+/–^* and *Tet2^–/–^* mice. (**G**) Percentage of LSK cells in the Lin^–^ bone marrow cells of moribund *Ep300*Δ*/*Δ*Tet2^+/–^* and *Ep300*Δ*/*Δ*Tet2^–/–^* mice and age-matched *Tet2^+/–^* and *Tet2^–/–^* mice. (**H**) Percentage of Mac-1^+^ cells in the bone marrow of moribund *Ep300*Δ*/*Δ*Tet2^+/–^* and *Ep300*Δ*/*Δ*Tet2^–/–^* mice and age-matched *Tet2^+/–^* and *Tet2^–/–^* mice. (**I**) Percentage of B220^+^ cells in the bone marrow of moribund *Ep300*Δ*/*Δ*Tet2^+/–^* and *Ep300*Δ*/*Δ*Tet2^–/–^* mice and age-matched Tet2^+/–^ and *Tet2^–/–^* mice. (**J**) Kaplan-Meier survival curve after poly(I:C) injections for each genotype (WT, *Ep300*Δ*/*Δ, *Tet2^+/–^*, *Ep300*Δ*/*Δ*Tet2^+/–^*, *Tet2^–/–^*, and *Ep300*Δ*/*Δ*Tet2^–/–^*) in transplantation models. *P* values were determined using a 2-tailed Student’s *t* test for samples of unequal variance. LSK, lineage^–^ Sca1^+^c-Kit^+^.

The loss of p300 in *Tet2-*deficient mice greatly accelerated disease development and significantly shortened their survival (Figure 1A). The moribund *Ep300*Δ*/*Δ*Tet2^–/–^* and *Ep300*Δ*/*Δ*Tet2^+/–^* mice showed an increased WBC count (Figure 1B) and splenomegaly and granulocytic sarcomas (Figure 1C and Supplemental Figure 1C). Increased blasts in the peripheral blood and bone marrow and splenic infiltration were also observed in *Ep300*Δ*/*Δ*Tet2^–/–^* and *Ep300*Δ*/*Δ*Tet2^+/–^* mice (Figure 1D), suggesting the onset of leukemia. The flow cytometry data confirmed that the loss of p300 increased the frequencies of c-Kit^+^ cells and lineage^–^ Sca1^+^c-Kit^+^ (LSK) cells in the bone marrow (Figure 1, E–G); we also found an increased percentage of myeloid cells (Mac1^+^; Figure 1H) and a decreased percentage of B cells (B220^+^; Figure 1I). We found no difference in the RBC count, hemoglobin, platelet count, or the percentage of T cells (CD3^+^) in the bone marrow of the various moribund mice (Supplemental Figure 1, D–G). The results from the transplantation models further confirmed the shortened survival and accelerated leukemia development (Figure 1J). In contrast, the deletion of p300 in WT mice did not alter hematopoiesis significantly (Figure 1 and Supplemental Figure 1). Taken together, our in vivo results indicate that the loss of p300 in the context of monoallelic or biallelic loss of *Tet2* accelerated disease progression and the onset of leukemia.

### The loss of p300 promoted the proliferation and self-renewal of Tet2-deficient HSPCs.

**Figure 2:**
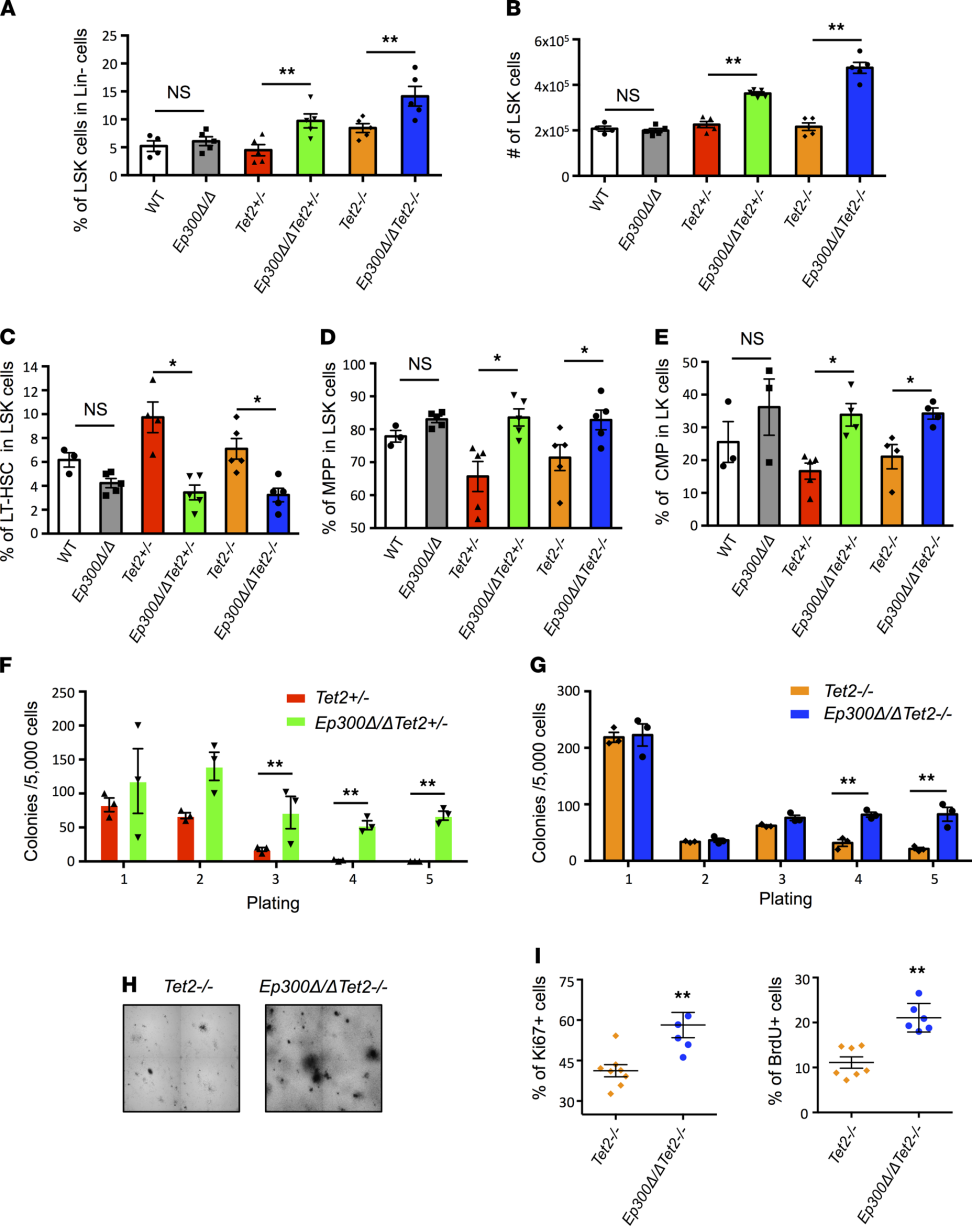
Loss of p300 enhances the proliferation and self-renewal of *Tet2*-deficient HSPCs. (**A**) Percentage of LSK cells in Lin^–^ bone marrow cells of the indicated mice 2 weeks after poly(I:C) administration. (**B**) Absolute number of LSK cells in the bone marrow of the indicated mice 2 weeks after poly(I:C) administration. (**C** and **D**) Percentage of LT-HSCs (**C**) and MPPs (**D**) in the LSK cells from the bone marrow of indicated mice 2 weeks after poly(I:C) administration (LT-HSCs, MPPs). (**E**) Percentage of CMP in the LK cells from the bone marrow of indicated mice 2 weeks after poly(I:C) administration (CMP, LK). (**F**) Number of colonies per 5000 cells seeded during serial replating of bone marrow cells from *Tet2^+/–^* and *Ep300*Δ*/*Δ*Tet2^+/–^* mice. (**G**) Number of colonies per 5000 cells seeded during serial replating of bone marrow cells isolated from *Tet2^–/–^* and *Ep300*Δ*/*Δ*Tet2^–/–^* mice. (**H**) Representative colony morphology in the 5th replating of bone marrow cells from *Tet2^–/–^* and *Ep300*Δ*/*Δ*Tet2^–/–^* mice. (**I**) Percentage of Ki67^+^ and BrdU^+^ cells in HSPCs from *Ep300*Δ*/*Δ*Tet2^–/–^* and *Tet2^–/–^* mice 2 weeks after poly(I:C) injections. *P* values were determined using 2-tailed Student’s *t* tests for samples of unequal variance. HSPCs, hematopoietic stem and progenitor cells; LSK, lineage^–^ Sca1^+^c-Kit^+^; LT-HSCs, long-term HSC; MPPs, multipotent hematopoietic progenitors; CMP, common myeloid progenitor, LK, Lin^–^ c-Kit^+^.

To further examine the initial consequences of p300 loss on hematopoiesis in *Tet2-*deficient mice, we analyzed HSPC numbers and function 2 weeks after *Ep300* deletion. As shown in Figure 2, A and B, and Supplemental Figure 2A, the frequency and absolute number of HSPCs were significantly increased after *Ep300* deletion in both *Tet2^–/–^* and *Tet2^+/–^* mice, but not in the WT mice, suggesting that the early loss of p300 perturbed HSPC function in the context of *Tet2* deficiency. Further analysis revealed that p300 loss in *Tet2-*deficient mice decreased the frequency of long-term HSCs (LT-HSCs) and increased the frequencies of multipotent hematopoietic progenitors (MPPs) and common myeloid progenitors within the HSPC population (Figure 2, C–E, and Supplemental Figure 2, B and C). This suggests that the loss of p300 in *Tet2-*deficient mice impaired HSPC differentiation and enhanced skewing toward myeloid progenitors.

Next, we performed serial replating assays to investigate the self-renewal and proliferation of *Tet2*-deficient HSPC after p300 loss. As shown in Figure 2F, the loss of p300 enhanced the replating capacity of *Tet2*^+/–^ HSPCs, suggesting an enhanced self-renewal. Moreover, the loss of p300 increased the number of colonies generated by *Tet2*-null HSPCs, indicating an enhanced proliferation as well (Figure 2, G and H). In vivo Ki67 and BrdU assays showed increased frequencies of Ki67^+^ cells and BrdU^+^ cells in *Ep300*Δ*/*Δ*Tet2^–/–^* HSPCs compared with *Tet2^–/–^* HSPCs, further confirming the hyperproliferative phenotype of *Ep300*Δ*/*Δ*Tet2^–/–^* HSPCs (Figure 2I). Meanwhile, the deletion of p300 did not affect the self-renewal of normal HSPCs (Supplemental Figure 2D). Taken together, the in vivo and in vitro results demonstrate that the loss of p300 enhanced the self-renewal and proliferation of *Tet2-*deficient HSPCs, increasing the bone marrow HSPC pool and accelerating leukemia development of the doubly deficient mice.

### The loss of p300 reprogrammed the epigenetic landscape and transcription network to enhance the proliferation and leukemogenicity of Tet2-null HSPCs.

Because TET2 and p300 played cooperative roles in maintaining the epigenome and enhancer landscape of HSPCs (26, 27), and our phenotypic data indicate that the loss of p300 in *Tet2-*deficient mice accelerated MDS progression, we next examined the TET2-independent function of p300 in HSPCs by comparing the epigenetic and gene expression profile of *Tet2^–/–^* HSPCs, before and after p300 loss.

Because TET2 catalyzes the conversion of 5 mC to 5 hmC and promotes DNA demethylation, to determine whether further loss of p300 affects DNA hydroxymethylation status, we performed DNA hydroxymethylation profiling (5-hMeDIP-Seq). As expected, *Tet2*^–/–^ HSPCs showed a global reduction in 5-hmC–enriched regions, compared with WT HSPCs. The deletion of *Ep300* in WT HSPCs resulted in a modest loss of 5-hmC (Supplemental Figure 3A), possibly reflecting the previously reported effects of p300-mediated acetylation on TET2 stability and activity (24). However, the deletion of *Ep300* in *Tet2*-null HSPCs induced minimal changes on global DNA hydroxymethylation levels (Supplemental Figure 3A).

**Figure 3:**
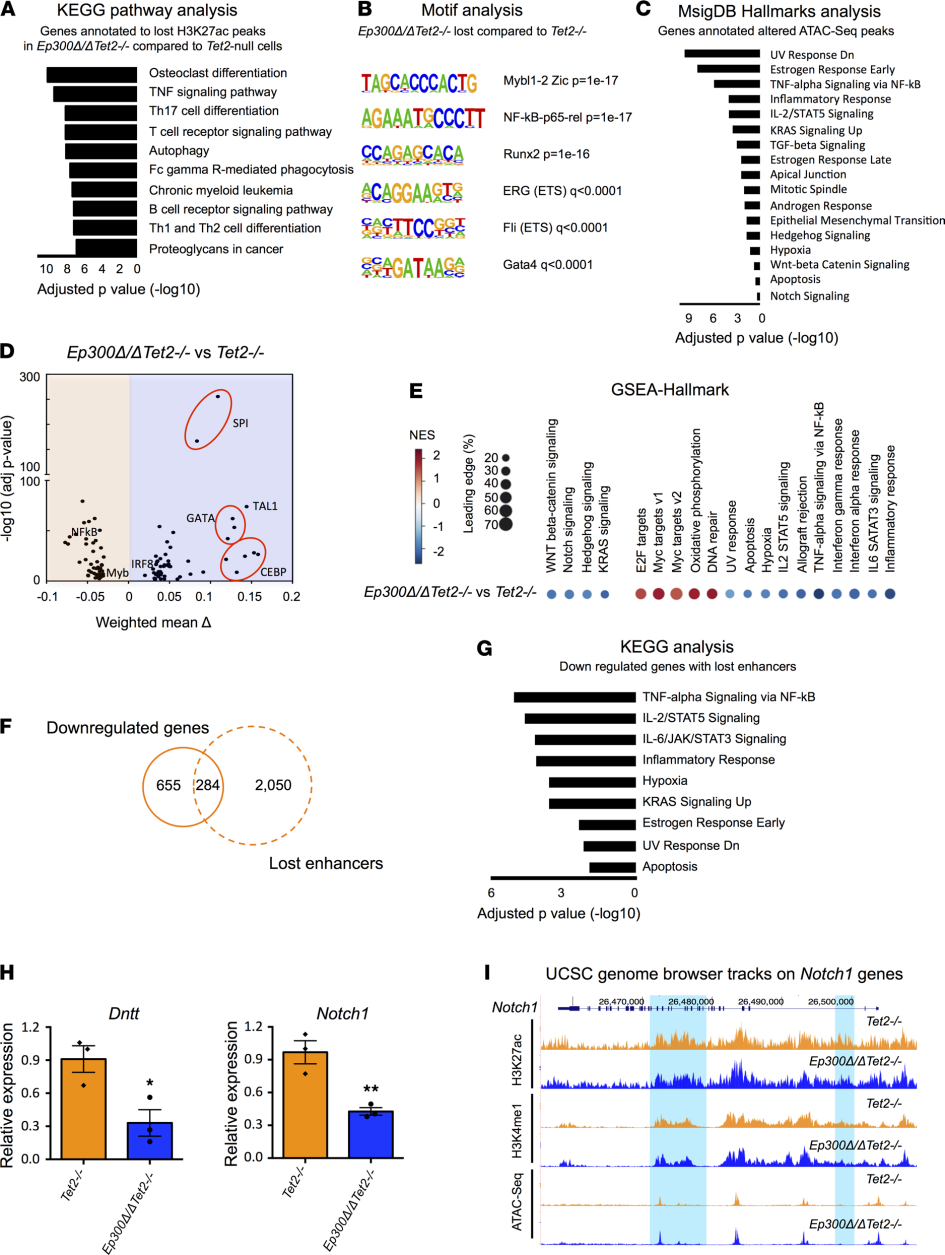
Loss of p300 rewires the epigenetic landscape and reprograms the transcriptome of *Tet2*-null HSPCs. (**A**) KEGG pathway enrichment analysis of the genes annotated to the H3K27Ac peaks lost in *Ep300*Δ*/*Δ*Tet2^–/–^* Lin^–^ cells compared with *Tet2^–/–^* Lin^–^ cells. (**B**) HOMER motif analysis of enhancers that lost in *Ep300*Δ*/*Δ*Tet2^–/–^* Lin^–^ cells compared with *Tet2^–/–^* Lin^–^ cells. (**C**) Enrichr-Hallmarks analysis of the genes annotated to the altered ATAC-Seq peaks in *Ep300*Δ*/*Δ*Tet2^–/–^* LSK cells compared with *Tet2^–/–^* LSK cells. (**D**) Volcano plot showing the differential transcription factor binding obtained from diffTF analysis of ATAC-Seq data in *Ep300*Δ*/*Δ*Tet2^–/–^* HSPCs compared with *Tet2^–/–^* HSPCs. Transcription factors that pass the significance threshold (adjusted *P* value < 0.01 and weighted mean difference greater that 0.03) are shown. (**E**) GSEA Hallmarks analysis of DE genes between *Ep300*Δ*/*Δ*Tet2^–/–^* and *Tet2^–/–^* HSPCs. (**F**) Overlap of the downregulated genes and the genes annotated to lost enhancers in *Ep300*Δ*/*Δ*Tet2^–/–^* cells compared with *Tet2^–/–^* cells. (**G**) Enrichr-KEGG analysis of the 284 genes identified in **F**. (**H**) Quantitative RT-PCR assays showing the expression of *Dntt* and *Notch1* in HSPCs from *Ep300*Δ*/*Δ*Tet2^–/–^* and *Tet2^–/–^* mice. (**I**) The UCSC genome browser tracks showing the ChIP-Seq and ATAC-Seq signal at *Notch1* gene locus. Highlighted in blue are 2 intronic enhancers of *Notch1*. Both tracks for each mark are adjusted to the same scale. *P* values were determined using 2-tailed Student’s *t* tests. HSPCs, hematopoietic stem and progenitor cells; LSK, lineage^–^ Sca1^+^c-Kit^+^; GSEA, Gene Set Enrichment Analysis; DE, differentially expressed.

Because p300 acetylates histone H3 at K27, a mark associated with active transcription and enhancer activity, we examined how p300 loss affects the distribution of H3K27ac and the enhancer landscape, in *Tet2*-null lineage^–^ (Lin^–^) bone marrow cells by performing ChIP-Seq analyses (Supplemental Figure 3B). In agreement with its role as transcriptional coactivator, the deletion of *Ep300* resulted in a genome-wide reduction of H3K27ac signal (Supplemental Figure 3B). The loss of p300 in *Tet2*-null cells led to a reduction in H3K27ac signal at 8950 genomic sites (Supplemental Figure 3C). Genes annotated to H3K27ac lost peaks were significantly enriched in pathways involved in differentiation, signal transduction, chronic myeloid leukemia, and TNF signaling (Figure 3A and Supplemental Table 1). We then identified active enhancers by overlapping H3K27ac and H3K4me1 ChIP-Seq peaks and analyzed the enhancer landscape of *Tet2*-null cells after p300 loss. Notably, p300 loss dramatically reprogrammed the enhancer landscape in *Tet2*-null cells, with a loss of 5204 active enhancers and a gain of 1732 active enhancers (Supplemental Figure 3D and Supplemental Table 2). Motif analyses of the altered enhancers showed enrichment for binding sites of key hematopoietic regulators including Ets, Runx, Gata, Irf8, NF-κB, and Myb family members (Figure 3B and Supplemental Figure 3D). These data reveal that p300 controlled the activity of a specific subset of enhancers in *Tet2*-deficient hematopoietic cells, enhancers that control the expression of genes involved in differentiation and leukemic transformation.

Because the regulation of gene expression by enhancers can be reflected in chromatin accessibility, we performed ATAC-Seq assays using HSPCs from *Ep300*Δ*/*Δ*Tet2^–/–^* and *Tet2^–/–^* mice (Supplemental Figure 3E). We found that p300 loss in *Tet2*-null HSPCs triggered alterations in chromatin accessibility, mostly at the intronic and intergenic regions (potential enhancer regions), and most often associated with a gain in ATAC-Seq signal (Supplemental Figure 3F). These additional epigenetic alterations may have occurred due to secondary or indirect effect of p300 loss in *Tet2^–/–^* cells. Of note, increases in chromatin accessibility were reported in *Tet2^–/–^* hematopoietic progenitors and AML cells, which were also suggested to be induced indirectly or secondarily by TET2 loss (25). Interestingly, changes (gains and losses) in ATAC-Seq signals in *Ep300*Δ*/*Δ*Tet2^–/–^* HSPCs (compared with *Tet2^–/–^* HSPCs) annotated to genes related to UV response, signal transduction, inflammatory response, and hypoxia (Figure 3C and Supplemental Table 3). To better identify potential TF binding patterns affected by p300 loss in the context of *Tet2^–/–^* cells, we analyzed our ATAC-Seq data using a recently developed computational method, *diffTF* (25, 28). The loss of p300 in *Tet2*-null HSPCs increased the binding accessibility of Spi1, Gata, Tal1, Irf, and Cebp TF families and decreased the binding accessibility of NF-κB and Myb family members (Figure 3D), indicating a major impact on transcription networks that control HSPC function.

To determine whether the alterations in the enhancer landscape induced by p300 loss in *Tet2^–/–^* HSPCs were associated with changes in gene expression, we performed RNA-Seq and Gene Set Enrichment Analysis (GSEA) using HSPCs isolated from WT, *Ep300*Δ*/*Δ, *Tet2^–/–^* and *Ep300*Δ*/*Δ*Tet2^–/–^* mice (Supplemental Figure 4, A–C, and Supplemental Table 4). The loss of p300 or Tet2 in normal HSPCs altered the expression of genes related to signaling pathways and proliferation, which is consistent with previous reports (29, 30). In *Tet2*-null HSPCs, p300 loss led 939 genes downregulated and 438 genes upregulated (Supplemental Figure 4B). GSEA analyses of the differentially expressed (DE) genes in *Tet2*-null HSPCs following *Ep300* deletion showed downregulation of genes that control hematopoietic differentiation (such as B cell and T cell differentiation), signal transduction (such as WNT, Notch and KRAS signaling), as well as inflammatory response (including IL2-STAT5 signaling and TNF-a signaling), and apoptosis (Figure 3E and Supplemental Figure 4, C and D). Notably, we observed a positive enrichment in gene pathways governing DNA repair and proliferation, such as E2F targets and Myc targets (Figure 3E). These results indicate that p300 loss triggered major alterations in the epigenomic landscape of *Tet2*^–/–^ HSPCs, resulting in altered gene expression patterns that affect their proliferation and differentiation, which is consistent with the hyperproliferative and enhanced self-renewal capacity of *Ep300*Δ*/*Δ*Tet2^–/–^* HSPCs (Figure 2).

To assess the association between the altered enhancer landscape and differential gene expression after p300 loss in *Tet2*-null hematopoiesis, we overlapped the DE genes and the genes that annotated to the altered enhancers. As shown in Figure 3F, out of 939 downregulated genes after p300 loss, 284 genes showed a loss of nearby active enhancers (i.e., loss of H3K27ac enrichment at intronic and intergenic regions marked by H3K4me1). These 284 genes are enriched for pathways controlling immune response, hypoxia, apoptosis, signal transduction, and differentiation (Figure 3G), and included important regulators of hematopoiesis such as *Dntt* and *Notch1* (Figure 3, H and I; refs. 31, 32). These changes likely reflect a direct effect of p300 on the enhancer accessibility and transcription output of *Tet2^–/–^* cells.

### The elevated Myb expression induced by p300 loss in Tet2-null HSPCs influenced the hyperproliferative phenotype.

**Figure 4:**
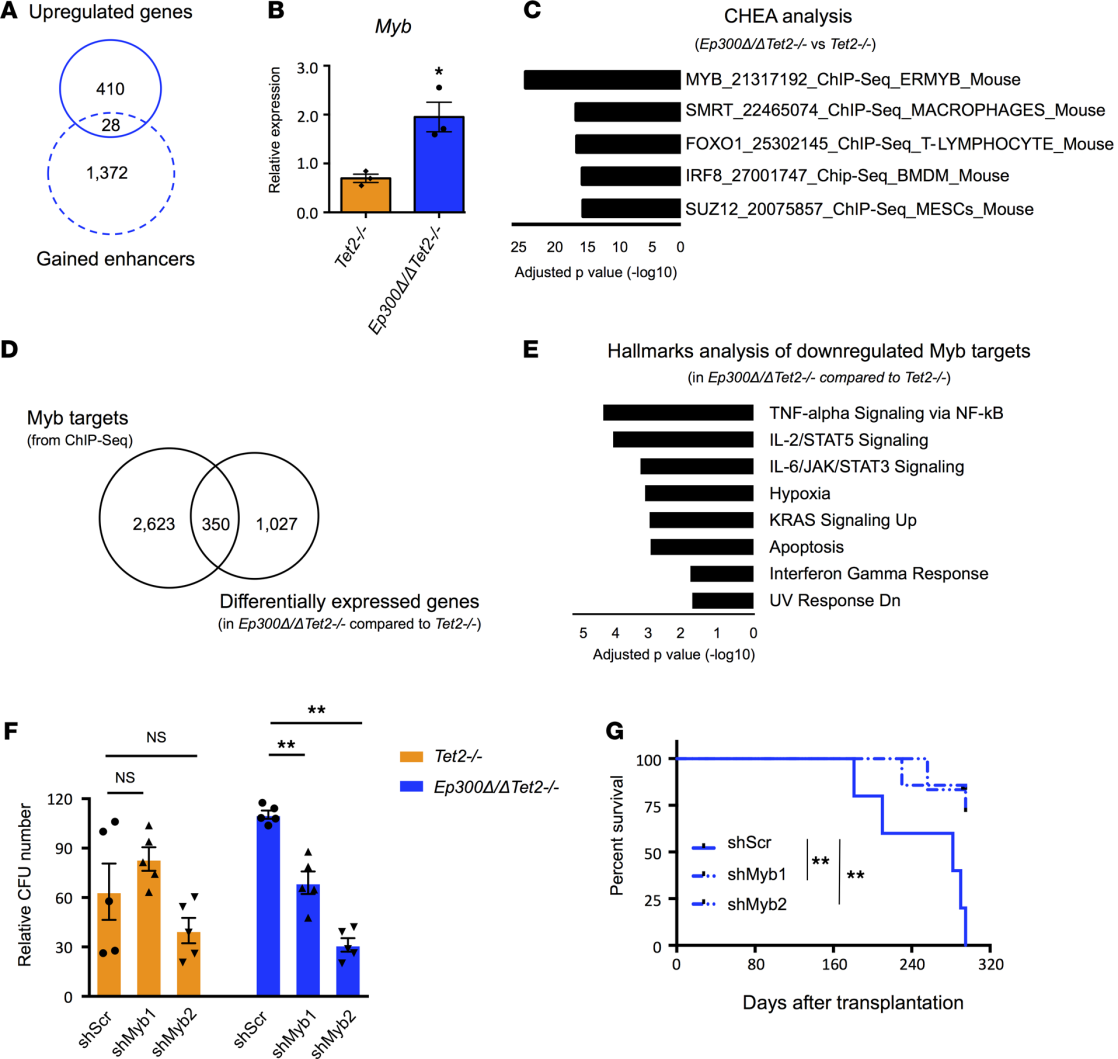
Enhanced proliferation and leukemogenicity of *Tet2*-null HSPCs after p300 loss are associated with increased *Myb* expression. (**A**) Overlap of the upregulated genes and the genes annotated to gained enhancers in *Ep300*Δ*/*Δ*Tet2^–/–^* cells compared with *Tet2^–/–^* cells. (**B**) Quantitative RT-PCR analysis of the expression levels of *Myb* in independent biological replicates of HSPCs from *Ep300*Δ*/*Δ*Tet2^–/–^* and *Tet2^–/–^* mice. (**C**) ChEA of the DE genes in *Ep300*Δ*/*Δ*Tet2^–/–^* HSPCs compared with *Tet2*-null HSPCs. (**D**) Overlap of the DE genes in *Ep300*Δ*/*Δ*Tet2^–/–^* HSPCs compared with *Tet2*-null HSPCs and Myb targets identified by ChIP-Seq. A total of 2974 Myb targets were identified from the ChIP-Seq data, and 350 of them were also DE genes. (**E**) Enrichr-Hallmarks analysis of the 303 downregulated genes identified as Myb targets. (**F**) Number of colonies obtained after 1 week of culture of 10,000 plated HSPCs from *Ep300*Δ*/*Δ*Tet2^–/–^* and *Tet2^–/–^* mice after *Myb* knockdown (*Myb*-KD) and cultured in methocult M3434 for 1 week. (**G**) Kaplan-Meier survival curves of mice receiving *Myb*-KD-leukemia cells from *Ep300*Δ*/*Δ*Tet2^–/–^* mice. *P* values were determined using a 2-tailed Student’s *t* test for **B** and a 2-way ANOVA test for **F**. HSPCs, hematopoietic stem and progenitor cells; ChEA, ChIP-X Enrichment analysis; DE, differentially expressed.

In addition to the downregulated genes, associated with a loss of nearby putative enhancers after p300 depletion in *Tet2*-null HSPCs, we observed a small portion of genes that gained expression and gained active enhancers (i.e., enrichment of H3K27ac at intronic and intergenic regions marked by H3K4me1) in *Ep300*Δ*/*Δ*Tet2^–/–^* HSPCs compared with *Tet2^–/–^* HSPCs (Figure 4A). Among these genes, we identified the *Myb* locus and the *Hoxb* gene cluster (Figure 4, A and B, and Supplemental Figure 5, A and B), which have both been directly associated with enhanced proliferation and oncogenicity of HSCs (33, 34). The ChIP-X Enrichment Analysis (ChEA) of the DE genes between *Ep300*Δ*/*Δ*Tet2^–/–^* and *Tet2^–/–^* HSPCs also revealed a significant enrichment for Myb target genes (Figure 4C). Thus, we hypothesized that the elevated expression of *Myb* triggered by p300 loss in *Tet2*-null HSPCs may have driven the changes in gene expression and subsequent alterations in HSPC function, including malignant transformation. To evaluate this hypothesis, we identified Myb target genes by performing ChIP-Seq analyses of Myb in WT Lin^–^ cells and compared them with the gene expression profile (Supplemental Table 5). Notably, 350 DE genes in *Ep300*Δ*/*Δ*Tet2^–/–^* HSPCs compared with *Tet2^–/–^* HSPCs were identified as targets of Myb (Figure 4D); of these 350 genes, 303 of them were downregulated and enriched in pathways related to signal transduction, immune response, and apoptosis (Figure 4E), verifying that changes in Myb expression and activity may have accounted for some of the key transcriptional alterations seen in *Tet2^–/–^* HSPCs after p300 loss.

Myb is known to promote the survival and proliferation of HSCs and leukemia stem cells (34–36); thus, we performed in vitro and in vivo assays, using *Myb*-depleted cells, to determine the impact of depleting *Myb* on the proliferation of HSPCs lacking p300 and TET2 (Supplemental Figure 5C). The depletion of *Myb* significantly attenuated the in vitro colony-forming capacity of *Ep300*Δ*/*Δ*Tet2^–/–^* HSPCs, but not *Tet2^–/–^* HSPCs (Figure 4F and Supplemental Figure 5D). We also depleted *Myb* in the leukemia cells from *Ep300*Δ*/*Δ*Tet2^–/–^* mice and performed transplantation assays. As shown in Figure 4G, mice receiving *Myb*-depleted cells showed prolonged survival, compared with the mice receiving scrambled shRNA-infected cells. These results suggest that the increased *Myb* expression caused by p300 loss contributed to the proliferation and oncogenicity of *Tet2*-null HSPCs.

### The loss of p300 accelerated disease progression and shortened survival in multiple MDS models.

**Figure 5:**
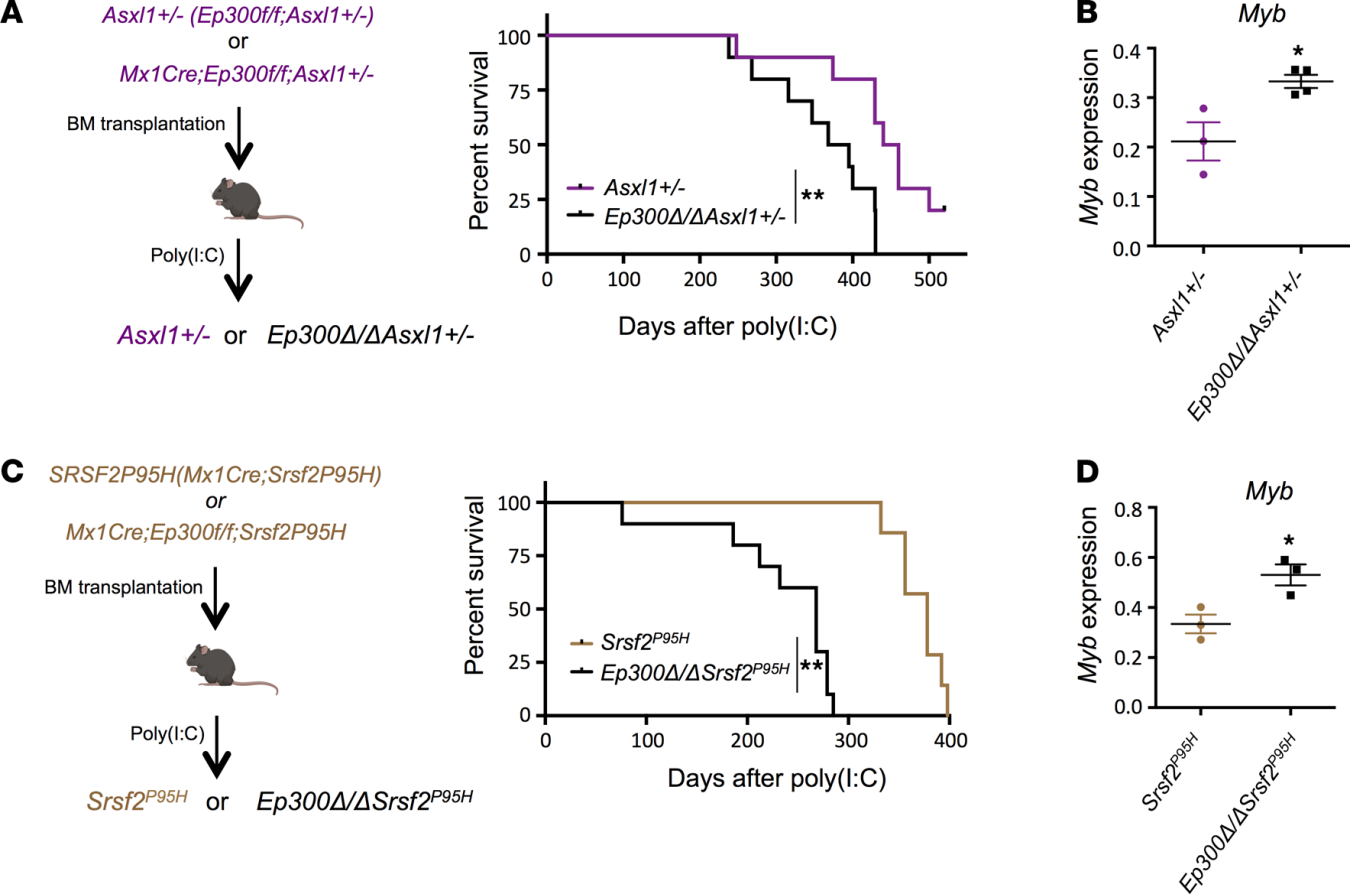
Loss of p300 in *Asxl1^+/–^* and *Srsf2^P95H^* mice increases the expression of *Myb* and shortens survival. (**A**) Experimental strategy to generate transplantation mouse models without or with hematopoietic-specific deletion of *Ep300* in *Asxl1^+/–^* genetic backgrounds. Kaplan-Meier curves show survival of the *Asxl1^+/–^* transplantation mouse models after *Ep300* deletion induced by poly(I:C) administration. Loss of p300 shortens the survival of *Asxl1^+/–^* mice. (**B**) Quantitative RT-PCR analysis of *Myb* mRNA expression in the bone marrow cells from *Ep300*Δ*/*Δ*Asxl1^+/–^* and *Asxl1^+/–^* mice. (**C**) Experimental strategy to generate transplantation mouse models without or with hematopoietic-specific deletion of *Ep300* in *Srsf2^P95H^* genetic backgrounds. Kaplan-Meier curves show survival of the *Srsf2^P95H^* transplantation mouse models after *Ep300* deletion induced by poly(I:C) administration. Loss of p300 shortens the survival of *Srsf2^P95H^* mice. (**D**) Quantitative RT-PCR analysis of *Myb* mRNA expression in the bone marrow cells from *Ep300*Δ*/*Δ*Srsf2^P95H^* and *Srsf2^P95H^* mice. *P* values were determined using 2-tailed Student’s *t* tests for **B** and **D**. The pictures of mice are from Biorender.

Although p300 loss in normal HSPCs has mild phenotypic consequences, its deletion in HSPCs lacking *Tet2* greatly alters HSPC behavior, triggering the development of AML. This suggests that p300 activity blocks oncogenic transformation of MDS/AML. We then examined the effects of p300 loss in other mouse models of MDS, driven by *Asxl1* haploinsufficiency or an *Srsf2* mutation (*Srsf2^P95H^*; ref. 3; Figure 5 and Supplemental Figure 5E). As shown in Figure 5, p300 loss shortened the survival of recipient mice, due to accelerated disease development, in both the *Asxl1^+/–^* and *Srsf2^P95H^* bone marrow transplantation models (Figure 5, A and C). The loss of p300 increased *Myb* expression in both *Asxl1^+/–^* and *Srsf2^P95H^* HSPCs (Figure 5, B and D), highlighting the consistent upregulation of *Myb* in the effect of p300 loss on MDS models driven by an array of mutations, which represent up to 70% of the genetic abnormalities found in MDS patients.

### Augmenting p300 KAT activity attenuated the enhanced self-renewal and proliferative capacity of Tet2-deficient HSPCs.

**Figure 6:**
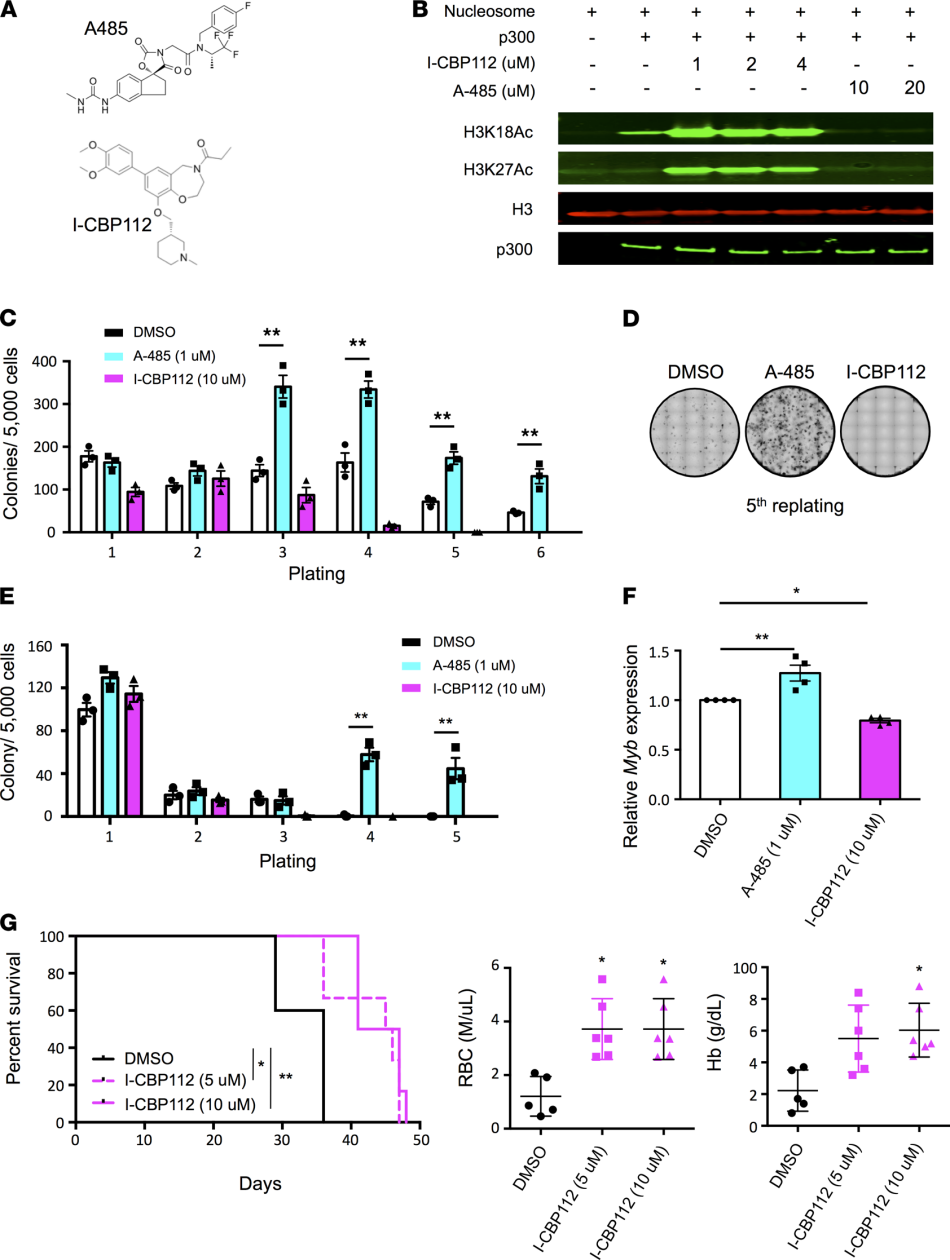
Augment of p300 KAT activity impairs the self-renewal and proliferation capacity of *Tet2*-deficient HSPCs. (**A**) Chemical structures of the p300 KAT inhibitor (A-485) and activator (I-CPB112) utilized in this study. (**B**) In vitro acetylation assay showing levels of acetylated H3K18 (H3K18Ac) and H3K27 (H3K27Ac) using polynucleosomes and recombinant p300 after treatment with the indicated doses of A-485 and I-CBP112. H3 total was used as loading control. (**C**) Number of colonies per 5000 cells seeded during serial replating of *Tet2^–/–^* cells treated with DMSO, A-485 (1 μM), or I-CBP112 (10 μM). (**D**) Representative morphology of colonies after 5 replatings in methocult M3434 in the indicated conditions. (**E**) Number of colonies per 5000 cells seeded during serial replating of *Tet2^+/–^* cells treated with DMSO, A-485 (1 μM), or I-CBP112 (10 μM). (**F**) Quantitative RT-PCR analysis of *Myb* mRNA expression in *Tet2^–/–^* LK cells after treatment with DMSO, A-485 (1 μM), or I-CBP112 (10 μM). (**G**) Kaplan-Meier survival curve of recipient mice after being transplanted with the bone marrow cells obtained from leukemic *Tet2^–/–^* mice treated ex vivo with DMSO or I-CBP112 (5 μM and 10 μM), and RBC count and Hb levels in peripheral blood 4 weeks after transplantation. *P* values were determined using 1-way ANOVA tests for **C** and **G** and 2-way ANOVA tests for **E** and **F**. HSPCs, hematopoietic stem and progenitor cells; LK, Lin^–^ c-Kit^+^; Hb, hemoglobin.

Given the tumor suppressor role of p300 in multiple MDS models, we next examined whether chemical modulation of p300 KAT activity using an inhibitor (A-485; refs. 37, 38) and an activator (I-CBP112; ref. 39) would similarly affect the self-renewal and proliferation of *Tet2-*deficient HSPCs (Figure 6A). First, we demonstrated that A-485 effectively inhibited, whereas I-CBP112 activated, the KAT activity of p300 toward histone H3K18 and H3K27 in the in vitro assays (Figure 6B). We then treated *Tet2-*deficient HSPCs with these compounds and performed serial replating assays. We found that A-485 enhanced the proliferation of *Tet2*^–/–^ HSPCs and the replating capacity of *Tet2^+/–^* HSPCs (Figure 6, C–E), phenocopying *Ep300* deletion in *Tet2*-deficient HSPCs. Conversely, I-CBP112 impaired the self-renewal and growth of both *Tet2*^–/–^ and *Tet2*^+/–^ HSPCs (Figure 6, C–E). Neither A-485 nor I-CBP112 influenced the self-renewal of normal HSPCs (Supplemental Figure 5F). Because increased *Myb* expression appears to be a p300-dependent event, we examined the expression of *Myb* in *Tet2^–/–^* LK (Lin^–^ and c-Kit^+^) cells after treatment in vitro with the p300 KAT modulators. We found increased *Myb* expression after A-485 treatment and decreased *Myb* expression after I-CBP112 treatment (Figure 6F), indicating that the regulation of *Myb* expression by p300 was dependent on its acetyltransferase activity.

We next evaluated the effect of I-CBP112 on the growth of *Tet2*-null leukemia cells in vivo. As shown in Figure 6G, mice receiving DMSO-treated *Tet2*-null leukemia cells developed aggressive AML with severe anemia (4 weeks after transplantation), whereas recipients of I-CBP112–treated *Tet2*-null leukemia cells showed improved survival with less severe anemia. Altogether, our data indicate that the KAT activity of p300 was required to limit the self-renewal, proliferation, and leukemogenicity of *Tet2*-null HSPCs, in part by controlling the expression of *Myb*. Small molecules that enhance p300 KAT activity could have been an effective therapeutic strategy for those hematopoietic malignancies that contain decreased TET2 activity.

## Discussion

We have uncovered a general role of p300 on the behavior of premalignant (*Tet2-*deficient, *ASXL1^+/–^*, and *SRSF2^P95H^*) HSPCs. In *Tet2-*deficient mice, the loss of p300 promoted the proliferation of HSPCs with myeloid skewing, resulting in an increased HSPC pool in the bone marrow and accelerated disease progression in vivo. Mechanistically, the lack of p300 activity disrupted the epigenetic landscape of *Tet2*-null HSPCs, as shown by the cumulative alterations in enhancer activity and chromatin accessibility observed in *Ep300*Δ*/*Δ*Tet2^–/–^* cells compared with *Tet2^–/–^* cells, highlighting an important function of p300 at the enhancers that control HSPC malignant transformation. Because p300 acetylates H3K27ac, which marks active enhancers, *Ep300* deletion triggered the loss of a large group of enhancers containing motifs for key hematopoietic regulators, such as NF-κB, Gata, Runx, Ets, and Myb family members (Figure 3B and Supplemental Figure 3D). These results aligned with our chromatin accessibility data, with motifs for the same hematopoietic TFs showing altered accessibility after p300 deletion (Figure 3D). As a consequence of this reprogrammed epigenome, the gene expression profile of *Tet2*-null HSPCs after p300 loss showed downregulation of pathways involved in B cell and T cell differentiation and signal transduction. For example, enhancer marks within the *Notch1* locus were decreased in *Tet2*-null cells by *Ep300* deletion and gene expression was also decreased. Silenced Notch signaling pathway has been directly linked to MLL-AF9–driven acute leukemogenesis (40). Our results provide another link between downregulated Notch1 signaling and cancer cells with diminished TET2 function and suggest a therapeutic potential of Notch receptor agonists in this setting. Although p300 loss in *Tet2*-null HSPCs resulted in a large group of lost enhancers, we still observed a small group of gained enhancers in *Ep300*Δ*/*Δ*Tet2^–/–^* HSPCs, some of which were associated with increased expression of genes, such as the *Hoxb* gene cluster and Myb, which are related to HSPC stemness and proliferation (33, 34).

The impact of p300 on MDS hematopoiesis is multifactorial, but our mechanistic and biological data implicate Myb as a critical player in *Tet2*-null hematopoiesis. First, p300 loss reduced the accessibility of Myb to chromatin and the expression of a subset of Myb target genes. Second, p300 loss increased *Myb* expression and the accessibility of the Myb enhancer. Third, we found that the depletion of *Myb* attenuated the hyperproliferative phenotype of *Ep300*Δ*/*Δ*Tet2^–/–^* HSPCs. Consistent with our data, it has been reported that blocking the interaction between Myb and p300 increases HSC numbers, and impairs lymphopoiesis and erythropoiesis, in mice (41). Thus, p300 deletion may affect the function and protein-protein interactions of Myb, contributing to defects in differentiation and signaling pathways and the enhanced proliferation and leukemogenicity that characterizes *Tet2*-null HSPCs. Although further studies are needed to decipher the precise mechanisms whereby p300-dependent regulation of Myb controls MDS pathogenesis, our study further implicates Myb as a promising target for therapy in MDS or AML patients (42).

Many studies have characterized p300 KAT activity and function in various cell types, including the identification of the p300 acetylome (43) and the potential use of p300 KAT modulators (inhibitors and activators) in solid tumor and leukemia treatment (44). We found that a p300 KAT inhibitor promoted the proliferation of *Tet2*-null HSPCs, whereas a KAT activator impaired their self-renewal and proliferative capacity.

In summary, we found that the loss of p300 or its chemical inhibition induced (a) a profound reprogramming of the epigenome and transcriptome of premalignant HSPCs, leading to impaired differentiation and signal transduction; (b) a unique gene expression signature in *Tet2*-null HSPCs, related to enhanced proliferation and oncogenicity; (c) an increase in *Myb* expression, which directly amplifies malignant transformation of MDS HSPCs; (d) an accelerated disease progression in multiple MDS models. Lastly, we demonstrate the therapeutic potential of enhancing p300 catalytic activity to treat MDS/AML patients with deficient TET2 function, and potentially individuals with *Tet2*-mutant clonal hematopoiesis.

## Methods

### Mice.

*Ep300^fl/fl^* mice were provided by Paul Brindle (St. Jude Children’s Research Hospital Memphis, Tennessee). *Mx1-Cre* (003556), *Srsf2^P95H^* (028376) and C57BL/6.SJL (002014) mice were purchased from The Jackson Laboratory. *Tet2^–/–^* and *Asxl1^+/–^* mice were previously described (12, 45). *Mx1-Cre*; *Ep300^fl/fl^*; *Tet2*–deficient mice were generated by breeding *Ep300^fl/fl^* mice with *Mx1-Cre* mice and with *Tet2^–/–^* or *Tet2^+/–^* mice. Both male and female mice were used in this studies and all of the mice were in C57BL/6 background.

The mice genotyped as *Ep300^fl/fl^* (WT), *Mx1-Cre;Ep300^fl/fl^* (*Ep300*Δ*/*Δ), *Ep300^fl/fl^*;*Tet2^–/–^* (*Tet2^–/–^),* *Mx1-Cre;Ep300^fl/fl^,* and *Mx1-Cre;Ep300^fl/fl^*;*Tet2^–/–^* (*Ep300*Δ*/*Δ*Tet2^–/–^*) were injected with poly(I:C) (10 μg/g raw body weight; Invivogen, tlrl-pic-5) at 6 to 8 weeks old via i.p. every other day, for a total of 3 doses to induce the deletion of *Ep300*. Experiments were performed either 2 weeks after poly(I:C) injections or at the indicated times. Endpoint mice were analyzed when moribund. Peripheral blood, spleen, and bone marrow samples were collected from moribund mice and age-matched control mice.

In the transplantation model, donor bone marrow cells were collected from *Ep300^fl/fl^*; *Asxl1^+/–^* (*Asxl1^+/^*), *Mx1-Cre;Ep300^fl/fl^*;*Asxl1^+/–^* (*Ep300*Δ*/*Δ*Asxl1^+/–^*), *Mx1-Cre;Srsf2^P95H^* (Srsf2^P95H^), *Mx1-Cre;Ep300^fl/fl^Srsf2^P95H^* (*Ep300*Δ*/*Δ*Srsf2^P95H^*), *Ep300^fl/fl^*;*Tet2^–/–^* (*Tet2^–/–^*), and *Mx1-Cre;Ep300^fl/fl^*;*Tet2^–/–^* (*Ep300*Δ*/*Δ*Tet2^–/–^*) mice; 2 × 10^6^ bone marrow cells were injected into lethally irradiated recipient mice (C57BL/6.SJL mice); and poly(I:C) was administered i.p. into recipient mice 4 weeks after transplantation.

### Antibodies and reagents.

p300 (KAT3B) active human recombinant protein (GWB-PSF0ED) was purchased from GenWay Biotech. Anti–β-Actin antibody (sc-130656, Santa Cruz Biotechnology) was used as loading control for Western blot. Anti-H3K27ac polyclonal antibody (C15410196, Diagenode), anti-H3K18ac (07-354, MilliporeSigma), anti-H3K4me1 (C15410194, Diagenode), anti-H3K4me3 (C15410003-50, Diagenode), anti-H3 (ab10799, Abcam), and Anti-Myb (ab45150, Abcam) antibodies were used for ChIP-Seq assays. For flow analyses, PE anti-mouse CD150 (115904), Pacblue anti-mouse CD48 (103418), APC anti-mouse B220 (103212), PEcy7 anti-mouse Sca-1 (108114), PerCPcy5.5 anti-mouse Gr1 (108428) antibodies, and streptavidin APC-cy7 (405208) were purchased from Biolegend. The Biotin Mouse Lineage Panel (559971), Biotin Rat anti-mouse CD8a (553029), Biotin Rat Anti-Mouse CD4 (553728), Biotin Rat Anti-Mouse CD5 (553019), Biotin Rat Anti-Mouse CD19 (553784), Biotin Rat Anti-Mouse CD127 (555288), APC anti-mouse c-Kit (553356), PE anti-mouse CD16/32 (553145), APC anti-mouse Mac1 (550019), PE anti-mouse B220 (561878), APC-Cy7 anti-mouse CD3 (560590) antibodies, APC BrdU Flow kit (552598), and PE Mouse Anti-Ki-67 Set (556027) were purchased from BD Pharmingen. A-485 (6387/5) was purchased from Thermo Fisher Scientific. I-CPB112 (SML1134-5MG), EDTA-free Protease Inhibitor Cocktail (04693159001), and phosphatase inhibitor tablets (04906837001) were purchased from MilliporeSigma.

### Flow cytometry analysis and cell sorting.

Cells were stained with the indicated antibodies and analyzed by FACSCanto II cytometer (BD Biosciences) and sorted using FACSAria II cell sorter (Becton Dickinson). Data were analyzed using FACSDiva v8.0.1 (BD Biosciences) and FlowJo v10.1.

### Quantitative RT-PCR.

Total RNA was extracted from sorted HSPCs using the RNeasy Plus Micro kit (QIAGEN), and cDNA was synthesized using the iScript cDNA Synthesis kit (Bio-Rad). The following Taqman probes were purchased from Invitrogen: *Ep300* (Mm01310115_m1), *Myb* (Mm00501741_m1), *Dntt* (Mm00493500_m1), *Notch1* (Mm00627185_m1), and *Gapdh* (Mm99999915_g1) as housekeeping control. Quantitative RT-PCR was performed on an ABI 7500 real-time cycler.

### Serial replating/CFU assays.

Five thousand HSPCs were sorted from bone marrow samples 2 weeks after poly(I:C) injections and seeded in MethoCult GF M3434 medium (STEMCELL Technologies) in the presence or absence of the indicated concentrations of p300/CBP KAT inhibitor (A-485) or activator (I-CBP112). Colonies were scored on day 7 of culture and cells were replated weekly.

### Morphologic analysis.

Peripheral blood was collected from mice by retro-orbital bleeding and complete blood count was performed by an automated blood count (Hemavet System 950FS). Bone marrow cytopspin was performed after euthanized the mice. May-Grunwald–Giemsa staining was used for morphologic analysis of bone marrow cytospin. All images were taken using an inverted system microscope (Olympus).

### CFU assays with Myb knockdown in HSPCs.

HSPCs obtained from *Tet2^–/–^* and *Ep300*Δ*/*Δ*Tet2^–/–^* mice were cultured in X-VIVO medium with IL-3 (10 ng/mL), IL-6 (10 ng/mL), and stem cell factor (SCF; 100 ng/mL) overnight and infected with lentiviruses, which express shMyb or scrambled shRNA in a modified PLKO-RFP–expressing vector using a spinfection protocol. Three days after infection, RFP^+^ cells were sorted and seeded in MethoCult GF M3434 medium (STEMCELL Technologies, 10,000 cells/well). The colonies were counted after 7 days of culture on a STEMvision (STEMCELL Technologies).

### Transplantation of Ep300Δ/ΔTet2^–/–^ leukemia cells after Myb knockdown.

Leukemia cells obtained from *Ep300*Δ*/*Δ*Tet2^–/–^* mice were cultured in X-VIVO medium with IL-3 (10 ng/mL), IL-6 (10 ng/mL), and SCF (100 ng/mL) overnight and infected with lentiviruses, which express shMyb or scrambled shRNA in a modified PLKO-RFP–expressing vector using a spinfection protocol. Three days after infection, RFP^+^ cells were sorted and 250,000 cells/mouse were transplanted into lethally irradiated recipients with 500,000 normal bone marrow cells. The mice were monitored and euthanized when moribund.

### Ex vivo treatment of I-CBP112 in Tet2^–/–^ leukemia cells.

Leukemia cells from the bone marrow of *Tet2^–/–^* mice were cultured in vitro and treated with DMSO and I-CBP112 for 24 hours. Recipient mice (C57BL/6.SJL mice) were lethally irradiated and transplanted with 2 × 10^6^ treated *Tet2^–/–^* leukemia cells with 500,000 helper cells from WT mice (C57BL/6). Peripheral blood was collected at indicated time point.

### ChIP-Seq of histone modifications and analysis.

For ChIP of histone marks, 25 million bone marrow Lin^–^ cells obtained using the Direct Lineage Cell depletion kit (Miltenyi Biotec, 140-110-470) were crosslinked and processed with the ChIP-IT High Sensitivity kit (Active Motif 53040) following the manufacturer’s instructions. For ChIP, 5 μg of chromatin from Lin^–^ cells and 1 μg of H3K27ac, H3K27me3, H3K4me1, H3K4me3, or H3 antibody were combined with 10 ng of chromatin from Drosophila melanogaster S2 cells (Active Motif 53083) as spike-in control. ChIP-Seq libraries were generated using NEBNExt Ultra II DNA library prep kit for Illumina, and sequenced (paired-end, 75-bp reads) in a Nextseq 500 platform (Illumina).

### ChIP-Seq of Myb in Lin^–^ cells.

For ChIP-Seq of Myb, 25 million bone marrow Lin^–^ cells were obtained using the Direct Lineage Cell depletion kit (Miltenyi Biotec, 140-110-470). Cells pellets were suspended in buffer (0.1% SDS, 1% Triton, 1 mM EDTA, 16.7 mM Tris pH 8.1, 167 mM NaCl) and were sonicated for 14 cycles (30 seconds on/off) using the Diagenode Bioruptor Pico. Chromatin (1 μg) samples were used in each i.p. with 1.5 μg of antibody against Myb (Abcam, ab76009) or rabbit normal IgG (Cell Signaling, 2729S), and 20 μl of Protein A beads (NEB, S1425S). The washed beads were tagged with 2 μl of TDE1 from Illumina (FC-121-1030) in a 30 μl reaction. The beads were reversed cross-linked overnight at 65°C. The DNA was amplified for 12 cycles with Illumina primers. DNA sequencing was performed on Nextseq 500 for 75 cycles.

### ChIP-Seq and analysis.

Reads were trimmed for adapters using Cutadapt (v1.15 –nextseq-trim=20 –m 18). Fastq files were aligned to mouse mm10 using BWA (v0.7.13 aln -q 5 -l 32 -k 2) and deduplicated using Picard Mark Duplicates (v2.10.6). For H3K27ac, H3K4me3, H3K4me1, and Myb, peaks were determined by overlapping called narrow peaks by macs2 (v2.1.1.20160309) from pseudo-replicates with *q* < 0.05 with H3 used as background for histone marks and IgG used as background for Myb. Reads were quality filtered (Mapq > 30) and the mm10 blacklist (46). Shift and extension sizes were determined using phatompeakqualtools (v1.1). ChIPseeker (v1.14.0) and R (v3.4.1) were used for genomic annotation with promoters defined as a 3 kb window around the TSS (Ensembl). Bigwig signal files were generated by Macs2 as fold change over background. Peak overlaps were determined by merging peaks list from a given comparison and intersecting individual peak files to the combined list by at least 1 bp. Putative enhancers were determined by intersecting H3K27ac and H3K4me1 peak regions outside of promoter regions.

### RNA-Seq and analysis.

Lin^–^ bone marrow cells were enriched using the Direct Lineage Cell Depletion kit with LS columns (Miltenyi Biotec). Enriched Lin^–^ cells were stained with the mouse bone marrow biotin lineage panel, Streptavidin APC-cy7, APC-c-Kit, and PEcy7-Sca-1, and after staining all HSPCs were sorted. Total RNA was extracted from 1 × 10^5^ to 2 × 10^5^ sorted HSPCs using AllPrep DNA/RNA Plus micro kit (QIAGEN). The quality of RNA was evaluated on an Agilent Bioanalyzer (RIN>9). Ribosomal RNA was removed using the NEBNext rRNA Depletion kit (New England Biolabs E6310) following the manufacturer’s instructions, and RNA-Seq libraries were prepared using the Universal Plus mRNA-Seq kit (NuGEN Technologies). RNA-Seq libraries were sequenced on an Illumina HiSeq 2500 platform (paired-end, 100-bp reads) at the Sylvester Oncogenomics Shared Resource, to obtain more than 40 million paired-end reads per sample. Sequencing and analysis were performed of 3 independent biological replicates per genotyping group. Reads were aligned to the mm10 transcriptome and ERCC spike-in indexes using RSEM (v1.3.0) and STAR (v2.6.0c) with Gencode annotations. Differentially expressed genes were determined by DESeq2 (v1.18.1, Wald test, adjusted *P* < 0.05) after gene counts were corrected based on ERCC variances using RUVseq (v1.12.0). GSEA was performed using GSEA (v3.0) with genes preranked by the Wald statistic. Gene Ontology Analysis was performed using the Enrichr API and sorted by adjusted *P* value. Sample blind variance stabilized and ERCC-corrected log2 transformed gene counts and transcripts per million were used for plots with expression data. Heatmaps, GSEA and GO bar plots, and Venn diagrams were generated using python (v3.6.3), scipy (v1.1.0), matplotlib, and seaborn (v.0.8.1).

### 5-hMeDIP, sequencing and analysis.

Genomic DNA from sorted HSPCs was isolated using the AllPrep DNA/RNA Plus micro kit (QIAGEN). Approximately 200 ng of genomic DNA was sonicated followed by end-repair, A-tailing, and adaptor ligation using standard Illumina protocols. Ten percent of the volume was retained for Input. Immunoprecipitation with anti-5-hmC antibodies (1 μg/mL, Active Motif) was performed at 4°C overnight and immunoprecipitated DNA was recovered using protein G magnetic beads (Invitrogen). After proteinase K digestion, DNA was purified from all input and immunoprecipitated samples and amplified by PCR for library preparation. Libraries were sequenced by an Illumina HiSeq 2500 sequencer by the Cancer Genomic Core Facility at University of Miami. Reads were trimmed using cutadapt (–m25), aligned using bowtie2 (v2.0.5) to the mouse mm10 genome build, quality filtered (mapq > 30), and deduplicated. Peaks were called using macs2 (-g mm --nomodel --extsize 200 –B) and differential peaks were determined using macs2 bdgdiff from the intermediate lambda and pileup bed graphs generated from macs2 call peaks.

### ATAC-Seq and analysis.

HSPCs (200,000) were sorted from mouse bone marrow samples and cryopreserved in BAMBAKER Serum-free cell freezing medium (Wako Chemicals). Cells were defrosted and washed twice in PBS, and 50,000 viable HSPCs were then processed following the OMIM-ATAC-Seq protocol (47) with a dead cell precleanup step with DNase I (30 minutes at 37°C; Worthington catalog LS002007) followed by washing twice in cold PBS. Libraries were prepared utilizing 2×TD buffer (15027866, Illumina) and Robust Tn5 Transposase (EMQZ1422, Creative Biogene) and cleaned up with a Zymo DNA Clean and Concentrator-5 kit (catalog D4014). OMIM-ATAC-Seq libraries were amplified with a total of 11 PCR cycles with ATAC-Seq primers (48). Final libraries were quantified by Qubit and Bioanalyzer and sequenced on NOVASeq (paired-end, 100-bp reads) to obtain greater than 40 M reads/sample.

ATAC-Seq chromatin accessible regions were determined using ENCODE pipeline standards (https://github.com/ENCODE-DCC/atac-seq-pipeline; git commit 2b693ab). Briefly, sequencing indices were trimmed from merged fastq files using cutadapt (v1.9.1) and then aligned to mm10 using bowtie2 (v2.2.6). After deduplication, reads were tn5 shifted, replicate peaks were called, and fold enrichment signal files were generated using macs2 (v2.1.0). Final peaks were determined using Bedtools (v2.0.4) of true replicates. Accessibility heatmaps and profile plots were generated using deeptools (3.1.1).

Moreover, we applied DiffTF software (https://git.embl.de/grp-zaugg/diffTF), which was developed to identify the quantification of TF activity using ATAC-Seq data (25, 28). Briefly, the software identified the binding sites of 422 TFs using the mouse PWMs deposited in the database Hocomoco v10, and PWMScan. For each TF we then extracted the signal around each binding site (± 100 bp around the core motif) and calculated a fold change between 2 conditions. Welch’s 2-sample *t* test was used for significance of the differences in a comparison.

### Data availability.

5-hMeDIP-Seq, RNA-Seq, and ChIP-Seq data sets generated in this study have been deposited in the Gene Expression Omnibus database under accession number GSE145878.

### Statistics.

Kaplan-Meier curves were used for survival analysis. Graphpad Prism 6 was used for flow cytometry assays, complete blood count, colony-forming unit and serial replating assays, and quantitative RT-PCR assays. *P* values of less than 0.01 and 0.05 were considered significant. All graphs represent mean ± SEM. Survival curves were estimated using the Kaplan-Meier method and compared by the log-rank test. Two-tailed Student’s *t* test, one-way ANOVA tests, and two-way ANOVA tests were applied (indicated in each figure).

### Study approval.

All animal studies were conducted in compliance with *Guide for the Care and Use of Laboratory Animals* (National Academies Press, 2011) and were approved by the IACUC of the University of Miami (protocol no. 18-164).

## Author contributions

NM, GM, and SDN designed the experiments, and wrote and edited the manuscript. NM and GM conducted and analyzed the experiments, with input from SDN, PAC, LC, FCY, and MX. MTM, DLK, and MEF contributed to the 5-hMeDIP-Seq and conducted the analysis. GM, DLK, QY, and JS contributed to the ChIP-Seq, ATAC-Seq, and RNA-Seq experiments and conducted the analysis. HI and SC contributed to the pathology analysis. CM contributed to the in vitro acetylation assays, generated plasmids, and produced the lentiviral reagents. SD assisted with animal experiments. FL, JS, PJH, YX, and CC assisted with the Myb studies, BEZ and PAC contributed to the A-485 and I-CBP112 studies.

## Supplementary Material

Supplemental data

Supplemental tables 1-5

## Version 1 10/08/2021

Electronic publication

## References

[1] Goyama S, Kitamura T (2017). Epigenetics in normal and malignant hematopoiesis: an overview and update 2017. Cancer Sci.

[2] Sperling AS (2017). The genetics of myelodysplastic syndrome: from clonal haematopoiesis to secondary leukaemia. Nat Rev Cancer.

[3] Kennedy JA, Ebert BL (2017). Clinical implications of genetic mutations in myelodysplastic syndrome. J Clin Oncol.

[4] AACR Project GENIE Consortium (2017). AACR project GENIE: powering precision medicine through an international consortium. Cancer Discov.

[5] Smith AE (2010). Next-generation sequencing of the TET2 gene in 355 MDS and CMML patients reveals low-abundance mutant clones with early origins, but indicates no definite prognostic value. Blood.

[6] Saint-Martin C (2009). Analysis of the ten-eleven translocation 2 (TET2) gene in familial myeloproliferative neoplasms. Blood.

[7] Delhommeau F (2009). Mutation in TET2 in myeloid cancers. N Engl J Med.

[8] Pan F (2017). Tet2 loss leads to hypermutagenicity in haematopoietic stem/progenitor cells. Nat Commun.

[9] Moran-Crusio K (2011). Tet2 loss leads to increased hematopoietic stem cell self-renewal and myeloid transformation. Cancer Cell.

[10] Cimmino L (2017). Restoration of TET2 function blocks aberrant self-renewal and leukemia progression. Cell.

[11] Zhao Z (2016). The catalytic activity of TET2 is essential for its myeloid malignancy-suppressive function in hematopoietic stem/progenitor cells. Leukemia.

[12] Li Z (2011). Deletion of Tet2 in mice leads to dysregulated hematopoietic stem cells and subsequent development of myeloid malignancies. Blood.

[13] Zhang X (2016). Identification of focally amplified lineage-specific super-enhancers in human epithelial cancers. Nat Genet.

[14] Dancy BM, Cole PA (2015). Protein lysine acetylation by p300/CBP. Chem Rev.

[15] Rebel VI (2002). Distinct roles for CREB-binding protein and p300 in hematopoietic stem cell self-renewal. Proc Natl Acad Sci U S A.

[16] Kasper LH (2006). Conditional knockout mice reveal distinct functions for the global transcriptional coactivators CBP and p300 in T-cell development. Mol Cell Biol.

[17] Haery L (2014). Histone acetyltransferase-deficient p300 mutants in diffuse large B cell lymphoma have altered transcriptional regulatory activities and are required for optimal cell growth. Mol Cancer.

[18] Wang L (2011). The leukemogenicity of AML1-ETO is dependent on site-specific lysine acetylation. Science.

[19] Gao XN (2013). A histone acetyltransferase p300 inhibitor C646 induces cell cycle arrest and apoptosis selectively in AML1-ETO-positive AML cells. PLoS One.

[20] Kongkiatkamon S (2019). Molecular characterization of EP300 mutant myeloid neoplasia. Blood.

[21] Cheng G (2017). Loss of p300 accelerates MDS-associated leukemogenesis. Leukemia.

[22] Man N, Nimer SD (2018). p300 suppresses leukemia development in NUP98-HOXD13 driven myelodysplastic syndrome. Oncotarget.

[23] Taylor J (2020). Single-cell genomics reveals the genetic and molecular bases for escape from mutational epistasis in myeloid neoplasms. Blood.

[24] Zhang YW (2017). Acetylation enhances TET2 function in protecting against abnormal DNA methylation during oxidative stress. Mol Cell.

[25] Rasmussen KD (2019). TET2 binding to enhancers facilitates transcription factor recruitment in hematopoietic cells. Genome Res.

[26] Rasmussen KD (2015). Loss of TET2 in hematopoietic cells leads to DNA hypermethylation of active enhancers and induction of leukemogenesis. Genes Dev.

[27] Bowman RL, Levine RL (2017). TET2 in normal and malignant hematopoiesis. Cold Spring Harb Perspect Med.

[28] Berest I (2019). Quantification of differential transcription factor activity and multiomics-based classification into activators and repressors: diffTF. Cell Rep.

[29] Li R (2018). TET2 loss dysregulates the behavior of bone marrow mesenchymal stromal cells and accelerates Tet2-/--driven myeloid malignancy progression. Stem Cell Reports.

[30] Graf T (1992). Myb: a transcriptional activator linking proliferation and differentiation in hematopoietic cells. Curr Opin Genet Dev.

[31] Rothenberg EV (2014). Transcriptional control of early T and B cell developmental choices. Annu Rev Immunol.

[32] Radtke F (2004). Notch regulation of lymphocyte development and function. Nat Immunol.

[33] Qian P (2018). Retinoid-sensitive epigenetic regulation of the Hoxb cluster maintains normal hematopoiesis and inhibits leukemogenesis. Cell Stem Cell.

[34] Wang X (2018). MYB — a regulatory factor in hematopoiesis. Gene.

[35] Gonda TJ (1989). Activation of c-myb by carboxy-terminal truncation: relationship to transformation of murine haemopoietic cells in vitro. EMBO J.

[36] Patel G (1993). v-myb blocks granulocyte colony-stimulating factor-induced myeloid cell differentiation but not proliferation. Mol Cell Biol.

[37] Wang R (2018). Targeting lineage-specific MITF pathway in human melanoma cell lines by A-485, the selective small-molecule inhibitor of p300/CBP. Mol Cancer Ther.

[38] Lasko LM (2017). Discovery of a selective catalytic p300/CBP inhibitor that targets lineage-specific tumours. Nature.

[39] Zucconi BE (2016). Modulation of p300/CBP acetylation of nucleosomes by bromodomain ligand I-CBP112. Biochemistry.

[40] Lobry C (2013). Notch pathway activation targets AML-initiating cell homeostasis and differentiation. J Exp Med.

[41] Sandberg ML (2005). c-Myb and p300 regulate hematopoietic stem cell proliferation and differentiation. Dev Cell.

[42] Uttarkar S (2016). Targeting acute myeloid leukemia with a small molecule inhibitor of the Myb/p300 interaction. Blood.

[43] Weinert BT (2018). Time-resolved analysis reveals rapid dynamics and broad scope of the CBP/p300 acetylome. Cell.

[44] Benton CB (2017). Targeting histone acetylation: readers and writers in leukemia and cancer. Cancer J.

[45] Wang J (2014). Loss of Asxl1 leads to myelodysplastic syndrome-like disease in mice. Blood.

[46] Amemiya HM (2019). The ENCODE blacklist: identification of problematic regions of the genome. Sci Rep.

[47] Corces MR (2017). An improved ATAC-seq protocol reduces background and enables interrogation of frozen tissues. Nat Methods.

[48] Buenrostro JD (2015). ATAC-seq: a method for assaying chromatin accessibility genome-wide. Curr Protoc Mol Biol.

