# Supplementary material for: p300 suppresses the transition of myelodysplastic syndromes to acute myeloid leukemia
Source: JCI Insight. 2021 Oct 8;6(19):e138478. doi: 10.1172/jci.insight.138478 (PMC8525640; doi:10.1172/jci.insight.138478)
Supplement: Supplemental data [file jciinsight-6-138478-s304.pdf]

Supplemental materials

Supplementary figure legends

Supplementary Figure 1. Loss of p300 in *Tet2*-deficient mice accelerates the onset of leukemia and shortens survival.

(A) Experimental strategy to generate transplantation mouse models without or with *Ep300* deletion in WT, *Tet2-/-*, and *Tet2+/-* genetic backgrounds.

(B) Quantitative RT-PCR analysis of the expression levels of *Ep300* in the bone marrow cells 2 weeks after poly(I:C) administration.

(C) Representative H&E stained section and flow cytometry profile of a granulocytic sarcoma obtained from the uterus of an *Ep300Δ/ΔTet2-/-* mouse.

(D), (E) and (F) Red blood cell (RBC) (D), Hemoglobin (Hg) (E), and Platelet (PLT) (F) counts in peripheral blood at the endpoint of each indicated groups of mice. The WT, *Ep300Δ/Δ*, and *Tet2-/-* mice were age-matched to *Ep300Δ/ΔTet2-/-* mice and *Tet2+/-* mice were age-matched to *Ep300Δ/ΔTet2+/-* mice.

(G) Percentage of CD3+ cells in the bone marrow of moribund *Ep300Δ/ΔTet2+/-* and *Ep300Δ/ΔTet2-/-* mice and age-matched *Tet2+/-* and *Tet2-/-* mice.

p values were determined using a two-tailed Student's t test for samples of unequal variance.

Supplementary Figure 2. Loss of p300 enhances the proliferation and self-renewal capacity of *Tet2*-deficient HSPCs.

(A) Representative flow cytometry profiles of LSK populations in the Lin- bone marrow cells of WT, *Ep300Δ/Δ*, *Tet2-/-*, *Ep300Δ/ΔTet2-/-*, *Tet2+/-*, and *Ep300Δ/ΔTet2+/-* mice 2 weeks post poly(I:C) injections (LK, Lin- c-Kit+ Sca-1-).

(B) Percentage of ST-HSCs in LSK cells from the indicated mice 2 weeks after poly(I:C) administration (ST-HSCs: short-term HSCs).

(C) Percentage of MEP and GMP in LK cells from the indicated mice 2 weeks after poly(I:C) administration (MEP: megakaryocyte–erythroid progenitor cell, GMP: granulocyte-macrophage progenitor).

(D) Number of colonies per 5,000 cells seeded during serial replating of bone marrow cells isolated from WT and *Ep300Δ/Δ* mice.
p values were determined using a two-tailed Student's t test for samples of unequal variance.

Supplementary Figure 3. Loss of p300 rewires the epigenetic landscape of *Tet2*-null HSPCs.

(A) Bar plot showing the gain and lost 5-hmC peaks in each indicated comparison.

(B) Bar plots showing the number of ChIP-Seq peaks for H3K27ac, H3K27me3, H3K4me1, and H3K4me3 lost or gained in Lin- cells in the indicated comparisons.

(C) Overlap of H3K27ac ChIP-Seq peaks in Lin- cells from *Ep300Δ/ΔTet2-/-* and *Tet2-/-* mice.

(D) Overlap of active enhancers identified in *Tet2-/-* vs *Ep300Δ/ΔTet2-/-* Lin- cells, and transcription factor motif analysis (HOMER) of the 1,732 enhancers gained in *Ep300Δ/ΔTet2-/-* cells compared to *Tet2-/-* cells.

(E) Genomic distribution of ATAC-Seq peaks called in HSPCs from WT, *Ep300Δ/Δ*, *Tet2-/-*, and *Ep300Δ/ΔTet2-/-* mice.

(F) Pie chart and genomic distribution of ATAC-Seq peaks that are retained, lost or gained in *Ep300Δ/ΔTet2-/-* HSPCs compared to *Tet2-/-* HSPCs.

Supplementary Figure 4. Loss of p300 reprograms the gene transcription profile of *Tet2*-null HSPCs.

(A), (B), and (C) Heatmap showing the Z-scores of the differentially expressed (DE) genes from the HSPCs in the comparisons of *Ep300Δ/Δ* vs WT, *Tet2-/-* vs WT, and *Ep300Δ/ΔTet2-/-* vs *Tet2-/-*.

(D) Bar plot showing GSEA Hallmarks significantly enriched pathways (FDR<0.1) in *Ep300Δ/Δ* compared to WT HSPCs.

(E) Bar plot showing GSEA Hallmarks significantly enriched pathways (FDR<0.1) in *Tet2-/-* compared to WT HSPCs.

(F) and (G) Dot plot showing selected GSEA KEGG (C) and GSEA Gene Ontology-Biological Process categories (F) significantly enriched (NES>1; FDR<0.1) in *Ep300Δ/ΔTet2-/-* compared to *Tet2-/-* HSPCs. Dot size represents the % leading edge; dot color represents NES scaled from -2 to +2.

<u>Supplementary Figure 5. Enhanced proliferation and leukemogenicity of *Tet2*-null HSPCs after p300 loss are associated with increased *Myb* expression.</u>

(A) Average Z-score values obtained from RNA-Seq analyses showing the relative expression of *Hoxb* cluster genes in LSK cells from mice of the indicated genotypes.

(B) UCSC genome browser tracks showing the ChIP-Seq and ATAC-Seq signal at *Hoxb* cluster gene locus. Highlighted in blue is the enhancer (*DERARE*) location of the *Hoxb* gene cluster. Both tracks for each mark are adjusted to the same scale.

(C) Quantitative RT-PCR assays showing the expression of *Myb* in HSPCs from *Ep300Δ/ΔTet2-/-* and *Tet2-/-* mice after the depletion of *Myb*.

(D) Representative morphology of colonies obtained from HSPCs from *Ep300Δ/ΔTet2-/-* and *Tet2-/-* mice after depletion of *Myb* and cultured in methocult M3434 for 1 week.

(E) Quantitative RT-PCR assays showing the expression of *Ep300* in the bone marrow cells from *ASXL1+/-* and $SRSF2^{P95H}$ mice before and after *Ep300* deletion.

(F) Number of colonies per 5,000 cells seeded during serial replating of WT cells treated with DMSO, A-485 (1µM) or I-CBP112 (10µM).

p values were determined using a two-way ANOVA test for (C) and a two-tailed Student’s t test for (E).

<u>Supplementary table legends</u>

<u>Table 1. Genes annotated to altered H3K27ac peaks in *p300Δ/ΔTet2-/-* cells compared to *Tet2-/-* cells.</u>

Peaks were determined by overlapping called narrow peaks by macs2 (v2.1.1.20160309) from pseudo-replicates with $q<0.05$ with H3 used as background for histone marks. Altered peaks were identified as peaks that gained or lost H3K27ac enrichment in *p300Δ/ΔTet2-/-* cells compared to *Tet2-/-* cells. Altered peaks were then annotated to nearby genes (-/+ 2.5Kb from TSS).

Table 2. Genes annotated to altered enhancers in *p300Δ/ΔTet2-/-* cells compared to *Tet2-/-* cells.

Enhancers were identified as non-promoter regions of the genome enriched in H3K27ac and H3K4me1 peaks obtained by ChIP-Seq in the Lin- bone marrow cells. Lost enhancers correspond to enhancers that were called in *Tet2-/-* cells but not in *p300Δ/ΔTet2-/-* cells; Gained enhancers correspond to enhancers that were called in *p300Δ/ΔTet2-/-* cells but not in *Tet2-/-* cells. Genes were annotated to enhancers based on their closest location considering both upstream and downstream regions flanking the enhancers.

Table 3. Genes annotated to altered ATAC-Seq peaks in *p300Δ/ΔTet2-/-* HSPCs compared to *Tet2-/-* HSPCs.

ATAC-Seq chromatin accessible regions were determined using ENCODE pipeline standards. Peaks were identified using Bedtools (v2.0.4) and altered peaks correspond those with at least 2 fold difference in signal intensity in *p300Δ/ΔTet2-/-* compared to *Tet2-/-* LSK cells. Altered peaks were then annotated to nearby genes (-/+ 2.5Kb from TSS).

Table 4. Differentially expressed genes in *Ep300Δ/ΔTet2-/-* HSPCs compared to *Tet2-/-* HSPCs.

Differentially expressed genes were determined by DESeq2 (v1.18.1, Wald Test, p-adj < 0.05) after gene counts were corrected based on ERCC variances using RUVseq (v1.12.0).

Table 5. Genes annotated to Myb binding sites in WT Lin- bone marrow cells.

Myb ChIP-Seq peaks in Lin- bone marrow cells from WT mice were determined by overlapping called narrow peaks by macs2 (v2.1.1.20160309) from pseudo-replicates with q<0.05 with IgG used as background. Peaks were then annotated to nearby genes (-/+ 2.5Kb from TSS).

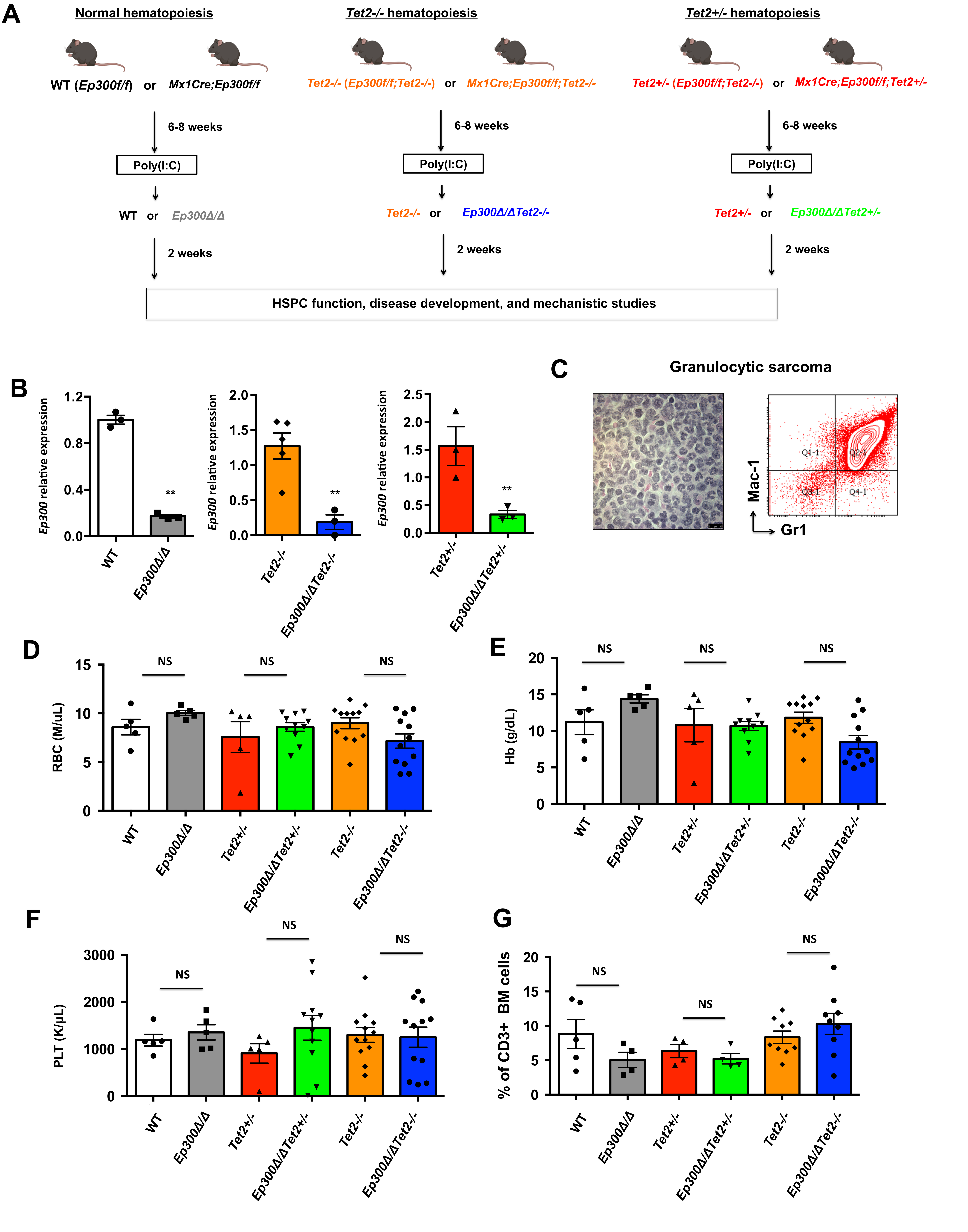

Supplementary Figure 1

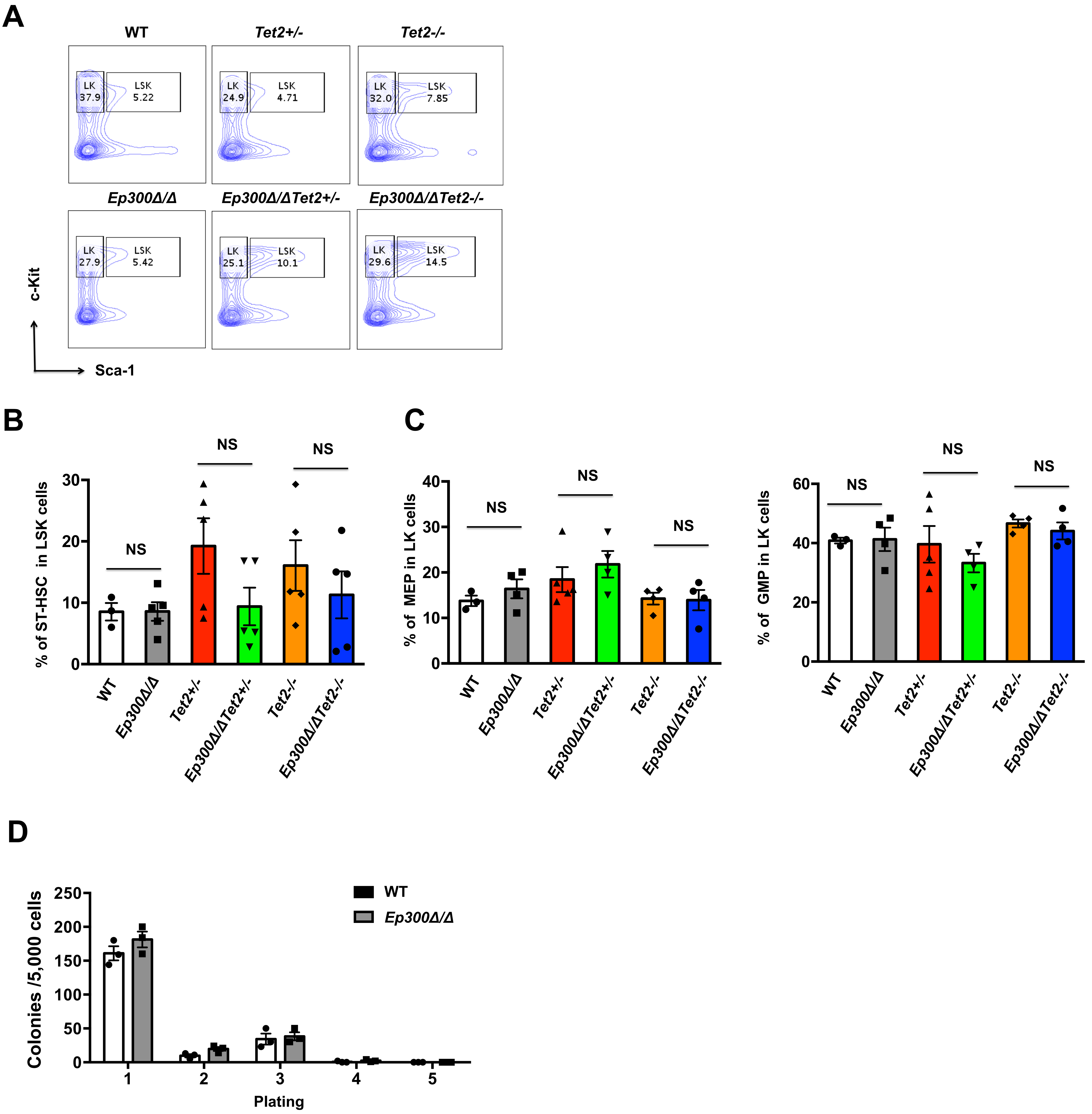

Supplementary Figure 2

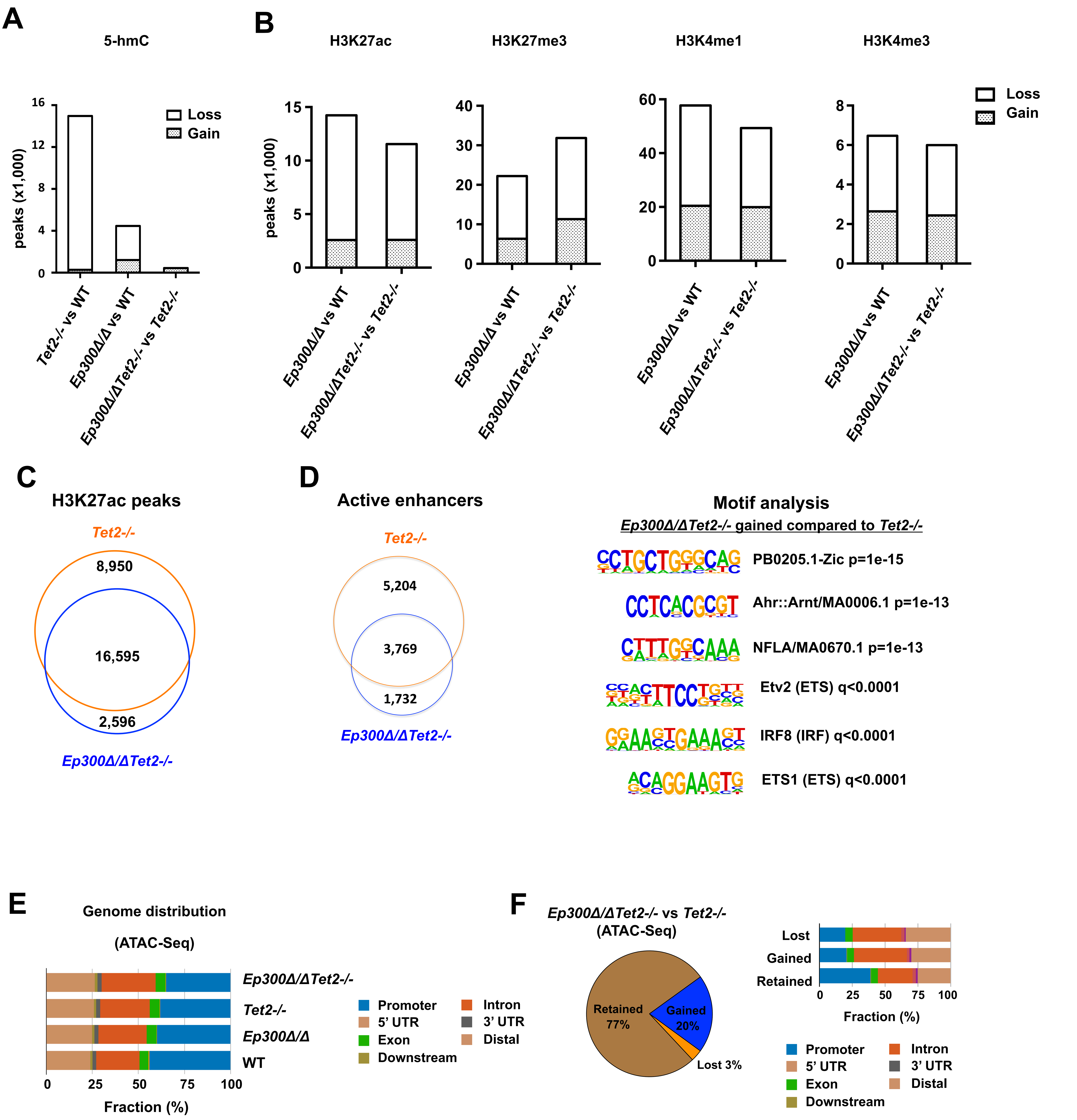

Supplementary Figure 3

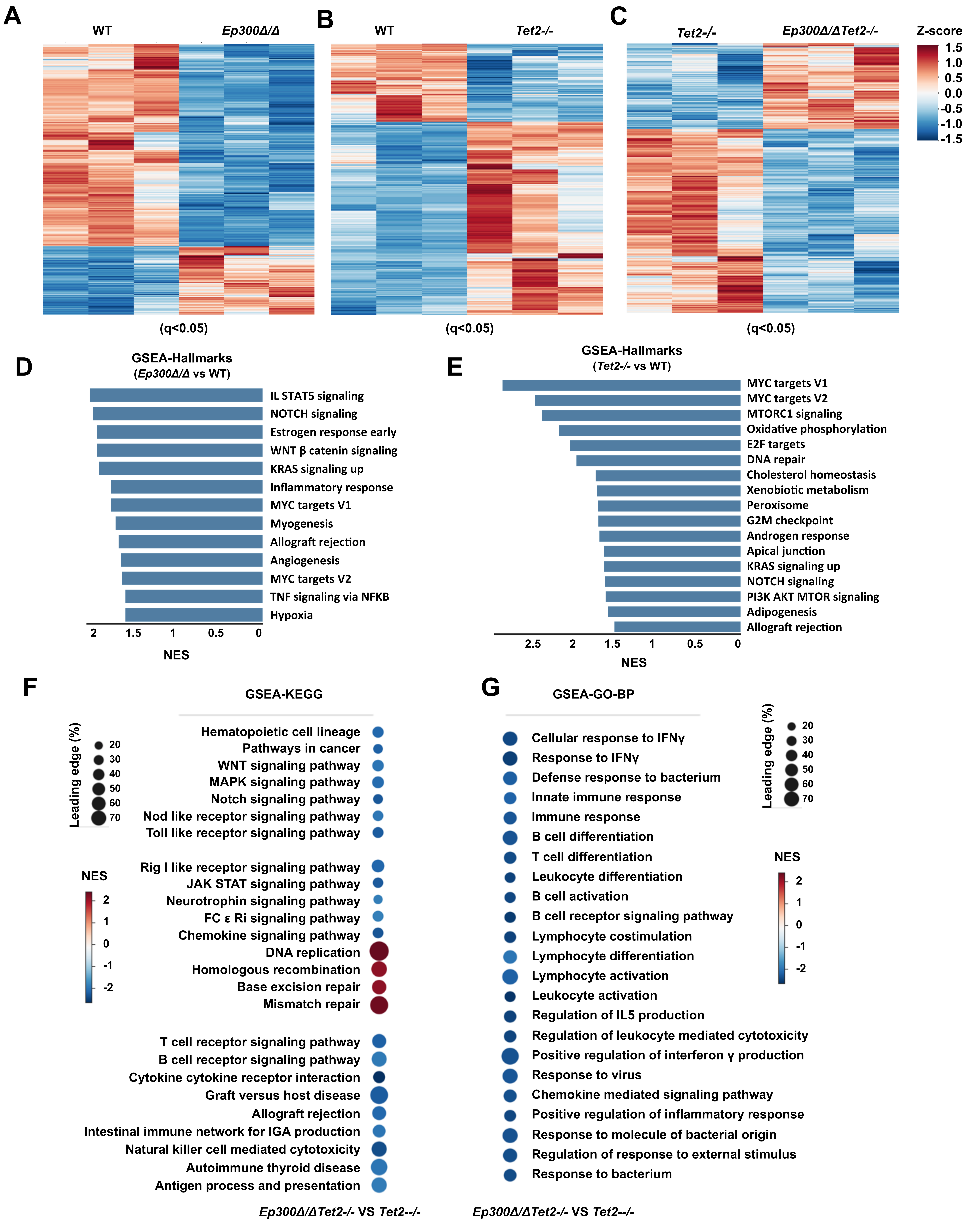

Supplementary Figure 4

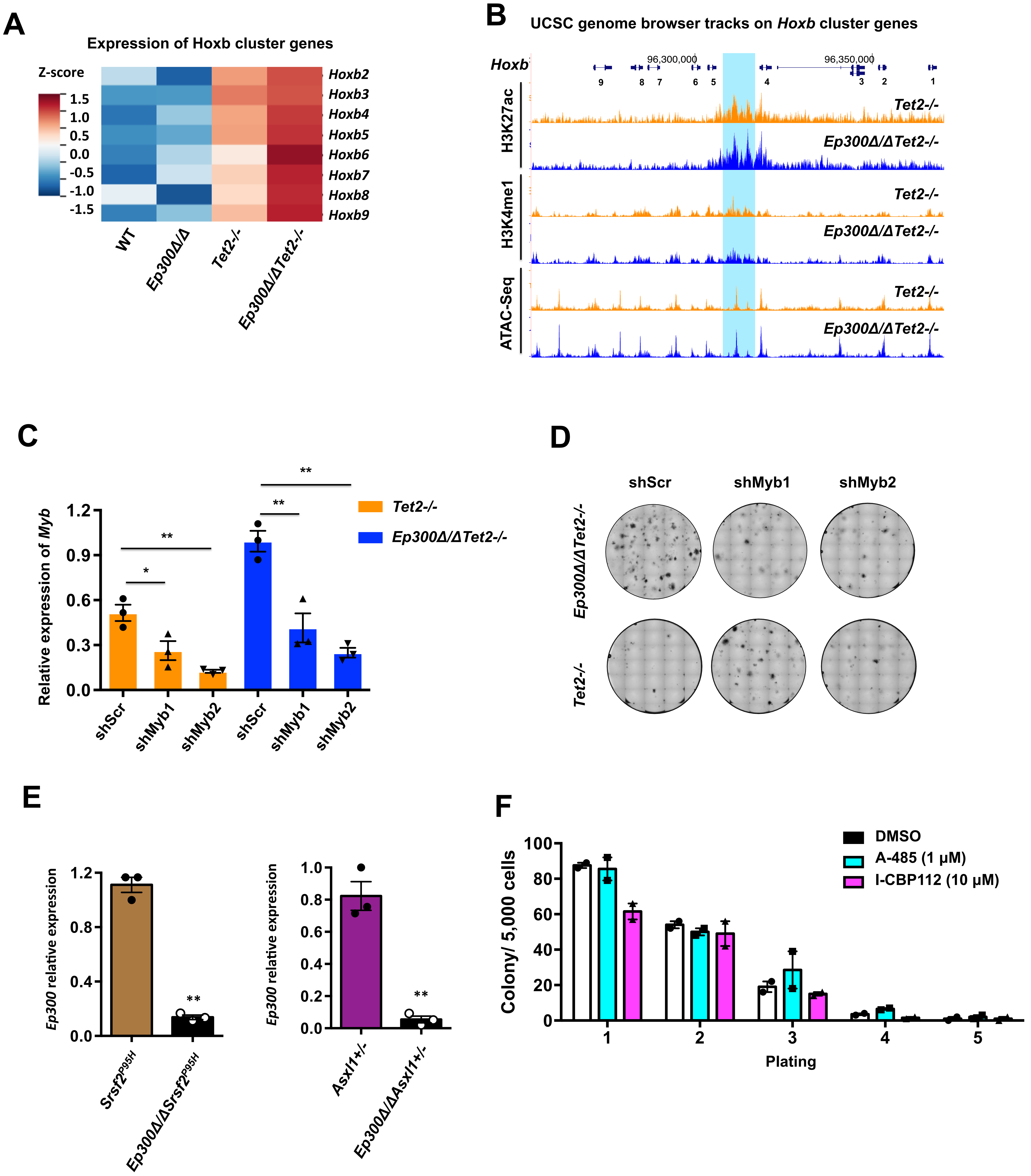

Supplementary Figure 5
